# An efficient numerical representation of genome sequence: natural vector with covariance component

**DOI:** 10.7717/peerj.13544

**Published:** 2022-06-16

**Authors:** Nan Sun, Xin Zhao, Stephen S.-T. Yau

**Affiliations:** 1 Department of Mathematical Sciences, Tsinghua University, Beijing, China; 2 Beijing Electronic Science and Technology Institute, Beijing, China; 3 Yanqi Lake Beijing Institute of Mathematical Sciences and Applications, Beijing, China

**Keywords:** Bacteria, Virus, Giant virus, Archaea, Fungi, Convex hull classification, The nearest neighbor classification, Phylogeny, Natural vector with covariance component

## Abstract

**Background:**

The characterization and comparison of microbial sequences, including archaea, bacteria, viruses and fungi, are very important to understand their evolutionary origin and the population relationship. Most methods are limited by the sequence length and lack of generality. The purpose of this study is to propose a general characterization method, and to study the classification and phylogeny of the existing datasets.

**Methods:**

We present a new alignment-free method to represent and compare biological sequences. By adding the covariance between each two nucleotides, the new 18-dimensional natural vector successfully describes 24,250 genomic sequences and 95,542 DNA barcode sequences. The new numerical representation is used to study the classification and phylogenetic relationship of microbial sequences.

**Results:**

First, the classification results validate that the six-dimensional covariance vector is necessary to characterize sequences. Then, the 18-dimensional natural vector is further used to conduct the similarity relationship between giant virus and archaea, bacteria, other viruses. The nearest distance calculation results reflect that the giant viruses are closer to bacteria in distribution of four nucleotides. The phylogenetic relationships of the three representative families, Mimiviridae, Pandoraviridae and Marsellieviridae from giant viruses are analyzed. The trees show that ten sequences of Mimiviridae are clustered with Pandoraviridae, and Mimiviridae is closer to the root of the tree than Marsellieviridae. The new developed alignment-free method can be computed very fast, which provides an effective numerical representation for the sequence of microorganisms.

## Introduction

With the increasingly close relationship between microorganisms and human beings, a deeper understanding of microorganisms becomes important (Wessner, Dupont & Charles, 2013). Comparing sequence similarity and inferring phylogenetic relationship is helpful to understand their properties, so as to reduce the harm of microorganisms and let them serve human beings better. Molecular biologists believe that similar sequences have similar functions. If the function of microbial species is known, the function of microorganisms with similar sequences can be inferred. Similar sequences can be obtained by sequence comparison. Two methods, alignment-free and alignment methods, are known to compare sequences. Traditional commonly used alignment approaches include MUSCLE (Edgar, 2004a; Edgar, 2004b), ClustalW (Larkin, Blackshields & Brown, 2007), and BLAST (Altschul et al., 1990; Altschul et al., 1997), but they are very time-consuming and require a lot of memory. Alignment-free methods are developed to overcome these limitations, such as chaos game representation (Jeffrey, 1990; Hatje & Kollmar, 2012), Fourier transform (Yin, Chen & Yau, 2014), information theory (Vinga, 2014; Almeida, 2014), k-mer theory (Dai, Yang & Wang, 2008; Leimeister & Morgenstern, 2014), *etc.* The alignment-free methods do not require the neutral theory assumption and can process a large number of microbial sequences.

Yau and his team proposed a powerful alignment-free method, a 12-dimensional natural vector, to describe the nucleotides distribution within the DNA sequence (Deng et al., 2011). This natural vector consists of the count, average position and central moment of each nucleotide. It has been successfully applied to many research fields, especially the clustering (Zhao, Tian & Yau, 2018; Zhao et al., 2019; Sun et al., 2021) and classification (He et al., 2020; Pei et al., 2020) of biological sequences. The 12-dimensional natural vector only considers the respective distribution of single nucleotide but ignores the relationship between the two nucleotides. This inspires us to extend the definition and further consider the correlation of each two nucleotides. There are four nucleotides for a sequence, and the new natural vector is 18-dimensional, of which the covariance component is six dimensional.

Microorganisms are diverse and live in every part of the biosphere, which mainly include archaea, bacteria, virus, fungi, and some other protozoa (Wessner, Dupont & Charles, 2013). As a vital part of microorganisms, the study of the giant virus has attracted much attention. Their evolutionary origin and the relationship with other viruses, bacteria and archaea remain controversial (Bichell, 2017). Giant viruses have extremely large genomes, some of which are even larger than bacterial genome (Ogata, Toyoda & Tomaru, 2009). Recently discovered giant viruses have even longer genome sequences and more encoding genes (Van Etten, 2011; Legendre, Arslan & Abergel, 2012). Researchers claim two hypotheses that they evolve either from small viruses or from very complex organisms. These enlighten us to analyze microbial sequences using our method.

In this article, we improve the 12-dimensional natural vector by defining the covariance between nucleotides, and add the six-dimensional covariance vector to the 12-dimensional vector. The 18-dimensional natural vector can effectively represent a sequence, and has been tested on five genomic sequence datasets and one DNA barcode sequence dataset. The results of convex hull classification show that the six-dimensional covariance vector is necessary to characterize sequences. The study of giant virus based on our method gives more stable results than other alignment-free methods. Our new 18-dimensional natural vector shows outstanding ability in classification and phylogeny of biological sequences.

## Materials and Methods

### Natural vector

Natural vector is a 12-dimensional numerical representation of a nucleotide sequence (Deng et al., 2011), which is defined as follows. Let $S = {s_1},\;{s_2},\;{s_3},\; \ldots {s_n}$ be a genomic sequence of length n, and $L = \left\{ {A,\;C,\;G,{\rm{T\;or\;U}}} \right\}$. For $k \;\in\; L$, the indicator functions ${w_k}( \cdot )\!\!:L \to \left\{ {0,1} \right\}$ is defined as:

(1) $$\matrix{ {{w_k}( {{s_i}} ) = \left\{ {\matrix{ {1,\;\;\;\;if\;{s_i} = k,} \cr {0,\;\;\;\;otherwise.} \cr } } \right.} \cr }$$

where ${s_i} \;\in\; L,\;i = 1,\;2,\;3,\; \ldots ,\;n.$ Let ${n_k} = \sum\nolimits_{i = 1}^n {{w_k}} ( {{s_i}} )$ denote the counts of nucleotide k in S, ${\mu _k} = \sum\nolimits_{i = 1}^n i {{{w_k}( {{s_i}} )} \over {{n_k}}}$ specify the average location of letter k, $D_j^k = \sum\nolimits_{i = 1}^n {{{{{( {i - {\mu _k}} )}^j}{w_k}( {{s_i}} )} \over {n_k^{j - 1}{n^{j - 1}}}}}$ be the j-th central moment of position of letter k. If $j = 2$, we get the traditional 12-dimensional natural vector:

(2) $$\matrix{ {( {{n_A},{n_C},{n_G},{n_T},{\mu _A},{\mu _C},{\mu _G},{\mu _T},D_2^A,D_2^C,D_2^G,D_2^T} )} \cr }$$

Here we give an example to calculate the vector. If the genomic sequence is ACGGTAGTCC, the indicator functions are ${w_A} = 1000010000,\;{w_C} = 0100000011,\;{w_G} = 0011001000,\;{w_T} = 0000100100$. Each component of the vector is calculated as follow:

• ${{\rm{n}}_{\rm{A}}} = 2,{{\rm{n}}_{\rm{C}}} = 3,{{\rm{n}}_{\rm{G}}} = 3,{{\rm{n}}_{\rm{T}}} = 2,$
• $\displaystyle {\mu _{\rm{A}}}{\rm{ = 1}} \cdot {{\rm{1}} \over {\rm{2}}}{\rm{\ +\ 6}} \cdot {{\rm{1}} \over {\rm{2}}}{\rm{\ =\ 3}}.{\rm{5}},$
• $\displaystyle {\mu _C} = 2 \cdot {1 \over 3} + 9 \cdot {1 \over 3} + 10 \cdot {1 \over 3} = 7,$
• $\displaystyle {\mu _{\rm{G}}}{\rm{ = 3}} \cdot {{\rm{1}} \over {\rm{3}}}{\rm{\ +\ 4}} \cdot {{\rm{1}} \over {\rm{3}}}{\rm{\ +\ 7}} \cdot {{\rm{1}} \over {\rm{3}}}{\rm{\ =\ 4}}.{\rm{67}},$
• $\displaystyle {\mu _T} = 5 \cdot {1 \over 2}\ +\ 8 \cdot {1 \over 2} = 6.5,$
• $\displaystyle D_2^A = {{{{( {1 - {7 \over 2}} )}^2}} \over {2 \cdot 10}} + {{{{( {6 - {7 \over 2}} )}^2}} \over {2 \cdot 10}} = 0.625,$
• $\displaystyle D_2^C = {{{{( {2 - 7} )}^2}} \over {3 \cdot 10}} + {{{{( {9 - 7} )}^2}} \over {3 \cdot 10}} + {{{{( {10 - 7} )}^2}} \over {3 \cdot 10}} = 1.27,$
• $\displaystyle D_2^G = {{{{( {3 - {{14} \over 3}} )}^2}} \over {3 \cdot 10}} + {{{{( {4 - {{14} \over 3}} )}^2}} \over {3 \cdot 10}} + {{{{( {7 - {{14} \over 3}} )}^2}} \over {3 \cdot 10}} = 0.289,$
• $\displaystyle D_2^T = {{{{( {5 - {{13} \over 2}} )}^2}} \over {2 \cdot 10}} + {{{{( {8 - {{13} \over 2}} )}^2}} \over {2 \cdot 10}} = 0.225.$

Then the 12-dimensional natural vector in formula (2) is:

$( {2,\;3,\;3,\;2,\;3.5,\;7,\;4.67,\;6.5,\;0.625,\;1.27,\;0.289,\;0.225} ).$

### A novel definition of covariance between nucleotides

The 12-dimensional natural vector definition reveals the respective distribution of four nucleotides, but ignores the relationship of each two nucleotides. We improve the 12-dimensional vector by defining the covariance between nucleotides. For the same genomic sequence $S = {s_1},\;{s_2},\;{s_3}, \ldots {s_n}$, and $L = \left\{ {A,\;C,\;G,\;{\rm{T\;or\;U}}} \right\}$, we redefine the indicator function, ${w_{kl}}( \cdot ) = {w_{lk}}( \cdot )\!\!:L \to \left\{ {0,1} \right\},\;k,\;l \;\in\; L\!\!:$

(3) $$\matrix{ {{w_{kl}}( {{s_i}} ) = {w_{lk}}( {{s_i}} ) = \left\{ {\matrix{ {1,\;\;\;\;if\ {s_i} = k\;or\;l,} \cr {0,\;\;\;\;\;\;\;otherwise.} \cr } } \right.} \cr }$$

And the covariance between k and l is defined as:

(4) $$\matrix{ {Cov( {k,l} ) = \sum\limits_{i = 1}^n \displaystyle{{{\left[ {i - {\mu _k}} \right]\left[ {i - {\mu _l}} \right]{w_{kl}}( {{s_i}} )} \over {n\sqrt {{n_k}} \sqrt {{n_l}} }}} .} \cr }$$

The above formula reflects the correlation relationship of the position of each two nucleotides. The beauty of the definition is $Cov( {k,k} ) = D_2^k$.

Thus, we get a novel 18-dimensional natural vector with covariance component:

(5) $$\eqalign{
  & ({n_A},\;{n_C},\;{n_G},\;{n_T},\;{\mu _A},\;{\mu _C},\;{\mu _G},{\mu _T},\;D_2^A,\;D_2^C,\;D_2^G,\;D_2^T,  \cr 
  & Cov(A,C),\;Cov(A,G),\;Cov(A,T),\;Cov(C,G),\;Cov(C,T),\;Cov(G,T)). \cr} $$

For the same sequence ACGGTAGTCC, the indicator functions are ${w_{AC}} = 1100010011,\;{{\rm{w}}_{{\rm{AG}}}} = 1011011000,\;{w_{AT}} = 1000110100,\;{w_{CG}} = 0111001011,  {w_{CT}} = 0100100111,\;{w_{GT}} = 0011101100.$ The corresponding components are calculated as follows:

• $\displaystyle Cov( {A,C} ) = \sum\nolimits_{i \;\in\; \left\{ {1,\;2,\;6,\;9,\;10} \right\}}^{} {{{\left[ {i - 3.5} \right]\left[ {i - 7} \right]} \over {10 \cdot \sqrt 2 \cdot \sqrt 3 }}} = 2.06,$
• $\displaystyle Cov( {A,G} ) = \sum\nolimits_{i \;\in\; \left\{ {1,\;3,\;4,\;6,\;7} \right\}}^{} {{{\left[ {i - 3.5} \right]\left[ {i - 4.67} \right]} \over {10 \cdot \sqrt 2 \cdot \sqrt 3 }}} = 0.864,$
• $\displaystyle Cov( {A,T} ) = \sum\nolimits_{i \;\in\; \left\{ {1,\;5,\;6,\;8} \right\}}^{} {{{\left[ {i - 3.5} \right]\left[ {i - 6.5} \right]} \over {10 \cdot \sqrt 2 \cdot \sqrt 2 }}} = 0.85,$
• $\displaystyle Cov( {C,G} ) = \sum\nolimits_{i \;\in\; \left\{ {2,\;3,\;4,\;7,\;9,\;10} \right\}}^{} {{{\left[ {i - 7} \right]\left[ {i - 4.67} \right]} \over {10 \cdot \sqrt 3 \cdot \sqrt 3 }}} = 1.56,$
• $\displaystyle Cov( {C,T} ) = \sum\nolimits_{i \;\in\; \left\{ {2,\;5,\;8,\;9,\;10} \right\}}^{} {{{\left[ {i - 7} \right]\left[ {i - 6.5} \right]} \over {10 \cdot \sqrt 3 \cdot \sqrt 2 }}} = 1.735,$
• $\displaystyle Cov( {G,T} ) = \sum\nolimits_{i \;\in\; \left\{ {3,\;4,\;5,\;7,\;8} \right\}}^{} {{{\left[ {i - 4.67} \right]\left[ {i - 6.5} \right]} \over {10 \cdot \sqrt 3 \cdot \sqrt 2 }}} = 0.54.$

Then the nucleotide distribution of sequence ACGGTAGTCC can be described by an 18-dimensional natural vector with covariance component:

$$(2,\;3,\;3,\;2,\;3.5,\;7,\;4.67,\;6.5,\;0.625,\;1.27,\;0.289,\;0.225,2.06,\;0.864,\;0.85,1.56,\;1.735,\;0.54).$$

The biological similarity between two sequences can be measured using the Euclidean distance of their corresponding 18-dimensional natural vectors, which is commonly used in our previous studies (Deng et al., 2011; Zhao, Tian & Yau, 2018; Zhao et al., 2019; Sun et al., 2021; He et al., 2020; Pei et al., 2020).

### Datasets and tools

The newly proposed alignment-free method is tested on six microorganism datasets, including the genome and gene sequences of archaea, bacteria, virus and fungi, as shown in Table 1. The genomes of archaea, bacteria, virus and fungi are downloaded from National Center for Biotechnology Information (NCBI). Giant viruses in this study are regarded as the viruses with genome size greater than 170 kb, which are collected to study their relationship with archaea, bacteria, and other viruses. This giant virus dataset is collected from two public databases (Giant virus toplist: https://pitgroup.org/giant-virus-toplist/; The largest known viral genomes (completely sequenced, >170 kb): http://www.giantvirus.org/2014-04-14top.html) and virus dataset (Viruses with genome size greater than 170 kb). In order to compare with the previous nucleotide covariance study (Zhao, Tian & Yau, 2018), we re-download DNA barcode of fungi from Barcode of Life Data System (BOLD) according to the dataset record (Zhao, Tian & Yau, 2018).

**Table 1:** The summary of the datasets in this study.

Dataset		Type	Number of sequences	Number of families	Database access link and description
1	Archaea	genomic sequence	298	20	ftp.ncbi.nih.gov/refseq/release/archaea/
2	Bacteria	genomic sequence	16,375	178	ftp.ncbi.nih.gov/refseq/release/bacteria/
3	Virus	genomic sequence	7,382	83	ftp.ncbi.nlm.nih.gov/genomes/Viruses
4	Fungi	genomic sequence	387	22	ftp.ncbi.nih.gov/refseq/release/fungi/
5	Giant virus	genomic sequence	677	16	Giant virus toplist: https://pitgroup.org/giant-virus-toplist/; The largest known viral genomes (completely sequenced, >170 kb): http://www.giantvirus.org/2014-04-14top.html; Reference sequences from Virus dataset, which genome size is over 170 kb, and the CDS counts are over 100.
6	Fungi	DNA barcode	95,542	467	Barcode of Life Data System (BOLD): http://www.barcodinglife.org

To ensure the reliability of the data, we remove three types of sequences from the original datasets: (1) sequences without family taxonomic information; (2) families with less three sequences. (3) sequences of plasmid for bacteria dataset. DNA barcode uniquely identifies species using a short fragment of DNA sequence from specific genes (iBOL, 2022). For this DNA barcode of fungi dataset, we only remain the sequences pertaining to internal transcribed spacer (ITS) region of fungi (Conrad, Keith & Sabine, 2012; Naturvetenskapliga, 2010). At last, there are 298 genomic sequences belonging to 20 families for archaea dataset, 16,375 genomic sequences belonging to 178 families for bacteria dataset, 7,382 genomic sequences belonging to 83 families for virus dataset, 387 genomic sequences belonging to 22 families for fungi genome dataset, 677 sequences belonging to 16 families for giant virus dataset, and 95,542 sequences belonging to 467 families for fungi DNA barcode dataset. All accession numbers can be found in Datas S1–S6.

All the programs in this article are written in MATLAB R2020a and run on the same laptop (MacBook Air, 1.8 GHz Intel Core i5, 8 GB 1,600 MHz DDR3).

### Convex hull principle for genomes

Convex hull principle for genomes has been demonstrated to be a wonderful classification tool (Zhao, Tian & Yau, 2018; Zhao et al., 2019; Sun et al., 2021). In Mathematics, the convex hull of a point set $A = \left\{ {{a_1},\;{a_2},\; \ldots ,{a_m}} \right\},{a_i} \;\in\; {R^k}$ is the minimal convex set that contains these m points, where ${R^k}$ is the k-dimensional Euclidean space, and ${a_i}$ is a k-dimensional point. The convex hull of finite set A is defined as the set of convex combinations of all points in A:

(6) $$\eqalign{ {CovA} =& \{ {\lambda _1}{a_1} + {\lambda _2}{a_2} + \ldots + {\lambda _m}{a_m}\!:\!{a_i} \;\in\; A,{\lambda _1} + {\lambda _2} + \ldots + \cr& {\lambda _m} = 1,{\lambda _i} \ge 0,\; \; \; \; i = 1,2, \ldots ,m\} }$$

The boundary of the convex hull is spanned by some points of A (vertexes), and the rest points of A are lying inside the hull. The convex hull is a convex polygon if all ${a_i}( {i = 1,2, \ldots ,m} )$ are two-dimensional vectors, and the convex hull is a convex polytope if all ${a_i}( {i = 1,2, \ldots ,m} )$ are high dimensional vectors. Particularly, triangles and tetrahedrons are convex hulls.

Here ${a_i}( {i = 1,2, \ldots ,m} )$ are 18-dimensional natural vectors, and they are divided into several classes (families). Intuitively, the distribution of nucleotides from the same family is similar, and the 18-dimensional points from the same families lie closely. The sequence is transformed into an 18-dimensional numerical vector first, and the points from the same family can form a convex hull. Convex hull principle states that convex hulls corresponding to different families are mutually disjoint (Zhao et al., 2019).

Linear programming (LP) can be used to check whether two convex hulls are disjoint (Sun et al., 2021). If $A = Cov\left\{ {{a_1},{a_2}, \ldots ,\!{a_m}} \right\}$ and $B = Cov\left\{ {{b_1},{b_2}, \ldots ,\!{b_n}} \right\}$ intersect, then the convex combination of these points satisfy the formula: $\sum\nolimits_{i = 1}^m {{\lambda _i}} {a_i} = \sum\nolimits_{j = 1}^n {{\beta _j}} {b_j}$, where $\sum\nolimits_{i = 1}^m {{\lambda _i}} = 1,\sum\nolimits_{j = 1}^n {{\beta _j}} = 1$. ${a_i}$ or ${b_j}$ is an 18-dimensional natural vector. It means that the following linear programming problem has a feasible solution (That is, there are non-zero coefficients $\left\{ {{\lambda _1},{\lambda _2}, \ldots ,{\lambda _m};{\beta _1},{\beta _2}, \ldots ,{\beta _n}} \right\}$ such that the minimum value of the optimization problem is 0):

(7) $$\displaystyle {\matrix{\min \;0. \\ {s.t.\sum\limits_{i = 1}^m {{\lambda _i}} {a_i} = \sum\limits_{j = 1}^n {{\beta _j}} {b_j}} \cr {\sum\limits_{i = 1}^m {{\lambda _i}} = 1,{\lambda _i} \ge 0,i = 1,2, \ldots ,m} \cr {\sum\limits_{j = 1}^n {{\beta _j}} = 1,{\beta _j} \ge 0,j = 1,2, \ldots ,n} \cr } }$$

The above problem can be implemented through *linprog* function built in MATLAB.

## Results

### Convex hull classification of archaea, bacteria, virus and fungi

The five datasets, archaea, bacteria, virus, fungi (genome), fungi (DNA barcode), are used for convex hull classification, and the necessity of adding extra six-dimensional nucleotide covariance component to 12-dimensional natural vector is verified. For each dataset, we first calculate the 12-dimensional or 18-dimensional natural vector to describe the distribution of each sequence, and construct convex hull for each family. Then the LP method is utilized to check whether the convex hulls of different families are disjoint. The comparison of classification performance between 12-dimensional natural vector and 18-dimensional natural vector is displayed in Table 2. For archaea dataset, there are 190 convex hull pairs, and 184 convex hull pairs are mutually disjoint in 12-dimensional space, while all pairs are disjoint in 18-dimensional space. For bacteria dataset, there are $C_{178}^2 = 15,\!753$ convex hull pairs, and 15,160 pairs are not intersected using 18-dimensional vector method. The non-intersection ratio in 18-dimensional space is 96.24%, which is larger than that in 12-dimensional space (92.46%). For virus dataset, there are 3,403 convex hull pairs. A total of 3,322 pairs are disjoint in 18-dimensional space, and 3,321 pairs are disjoint in 12-dimensional space. For both fungi (genome) and fungi (DNA barcode) datasets, the non-intersection ratios in 18-dimensional space are higher than those in 12-dimensional space. The results demonstrate that our new 18-dimensional natural vector performs better classification results than 12-dimensional natural vector. The 18-dimensional vector contains more sequence information because it considers the correlation relationships of the two nucleotides. The results of fungi (DNA barcode) dataset further show that covariance can be used not only for sequence classification at genome level, but also for sequence classification at gene sequence level.

**Table 2:** The number of disjoint convex hull pairs based on traditional 12-dimensional natural vector and our new 18-dimensional natural vector of adding the six-dimensional covariance vector. For 178 families of bacteria, there are 15,753 convex hull pairs. The number of disjoint convex hull pairs based on 18-dimensional natural vector is 15,160, which is more than that based on traditional 12-dimensional natural vectors (14,565). The other four datasets have similar conclusions. The results show that the six-dimensional covariance vector is necessary to characterize sequences. C_n_^k^ represents the combinatorial number.

Dataset	Convex hull pairs	12-dim	18-dim
Archaea	${\rm{C}}_{20}^2$ = 190	184	190
Bacteria	${\rm{C}}_{178}^2$ = 15,753	14,565	15,160
Virus	${\rm{C}}_{83}^2$ = 3,403	3,321	3,322
Fungi (Genome)	${\rm{C}}_{22}^2$ = 231	207	227
Fungi (DNA barcode)	${\rm{C}}_{467}^2$ = 108,811	75,237	88,719

To visualize the convex hull classification results, we use the traditional support vector machine method (SVM) to reduce the dimension (Sun et al., 2021). For two disjoint convex hulls in 18-dimensional space, there is a hyperplane to separate them:

(8) $$\matrix{ {{w^T}x + b = 0,} \cr }$$

**Figure 1:**
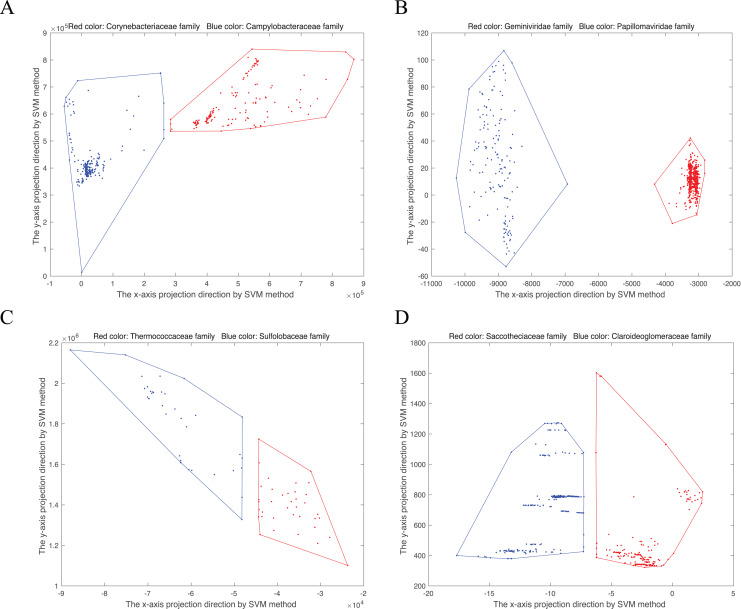
Convex hull pairs after dimension reduction by SVM method. (A) Convex hull pair constructed by two bacterial families, Corynebacteriaceae (243 points) and Campylobacteraceae (305 points). (B) Convex hull pair constructed by two virus families, Geminiviridae (565 points) and Papillomaviridae (154 points). (C) Convex hull pair constructed by two archaea families, Thermococcaceae (37 points) and Sulfolobaceae (51 points). (D) Convex hull pair constructed by two fungi (DNA barcode) families, Saccotheciaceae (274 points) and Claroideoglomeraceae (301 points).

$w = {( {{w_1},{w_2}, \ldots ,{w_k}} )^T}$ is the normal vector, b is the offset item. There is at least a vector $v$ perpendicular to $w$. For an 18-dimensional point, named $NV$ in the convex hull, the new point projected into two-dimensional space is $( {{w^T} \cdot NV,{v^T} \cdot NV} )$. Then the convex hull can be visualized in two-dimensional space. As shown in Fig. 1, we randomly select four disjoint convex hull pairs. Points from the same family gather together.

### Preliminary statistical analysis of the genomes of giant viruses

**Figure 2:**
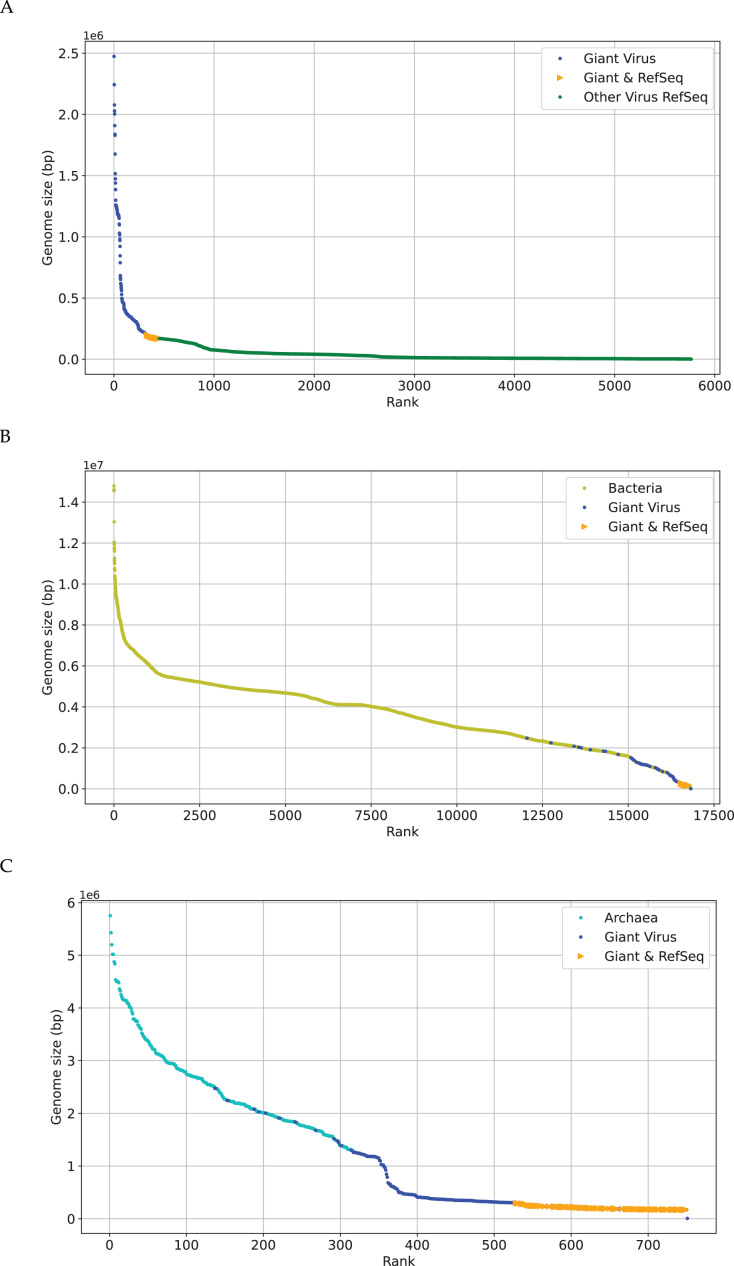
Genome size distribution of giant viruses. X-axis represents the number of the sequence, and Y-axis represents the genome size. Here “Giant Virus” only represents the sequences (genome size > 170 kb) of giant virus collected from two public databases. “Giant & RefSeq” represents the sequences (genome size > 170 kb) of giant virus collected from virus dataset. “Other Virus RefSeq” represents the rest sequences in virus dataset except for giant virus. (A) Genome size distribution of “Giant Virus”, “Giant & RefSeq” and “Other Virus RefSeq”. (B) Genome size distribution of bacteria, “Giant Virus” and “Giant & RefSeq”. (C) Genome size distribution of archaea, “Giant Virus” and “Giant & RefSeq”.

The research on phylogenetic relationship between giant virus and bacteria, archaea and other viruses is still active (Claverie & Abergel, 2013). The discovery of Mimivirus comes as a shock in biological field for a long time (Birtles, Rowbotham & Storey, 1997), and its genome is about 118 kbp (Didier, Stéphane & Catherine, 2004). Later viral genomes larger than 118 kbp started to accumulate quickly. This suggests that viruses with genomes larger than 118 kbp are not as exceptional as previously thought, especially Pandoravirus, which is discovered in recent years, with a length of more than 2,500 kbp (Philippe et al., 2013). Recent discoveries are not sufficient to erase the tremendous gap in genome size between giant viruses (Brandes & Linial, 2019), which is also reflected in Fig. 2. The x-axis represents the sequence number, the y-axis represents the genome size, and each dot represents a sequence. There are many gaps between genomes sizes of “Giant Virus” (blue color). Here “Giant Virus” only indicates the sequences of giant virus collected from two public databases (genome size > 170 kb). “Giant & RefSeq” indicates the sequences of giant virus collected from virus dataset (genome size > 170 kb). “Other Virus RefSeq” indicates the rest sequences in virus dataset except for giant virus. In Fig. 2A, there is a significant slope change at about 300 kbp, and the genome size of giant viruses is larger than that of other viruses (green color). In Fig. 2B, it is found that the genome sizes of several giant viruses and bacteria overlap at about 2,000 kbp, and the genome sizes of some viruses are larger than those of some bacteria. In Fig. 2C, the genome sizes of several giant viruses also overlap at about 2,000 kbp. Fig. 2 gives an intuitive understanding of the genome size relationship between giant virus and bacteria, archaea, other viruses. There is not yet any reason to believe that the genome size of giant viruses has reached the upper limit, and viruses with different genome lengths may emerge continuously (Claverie & Abergel, 2013). The origin of the giant virus still remains mysterious, so we can study it through the relationship between genomes of similar length.

**Figure 3:**
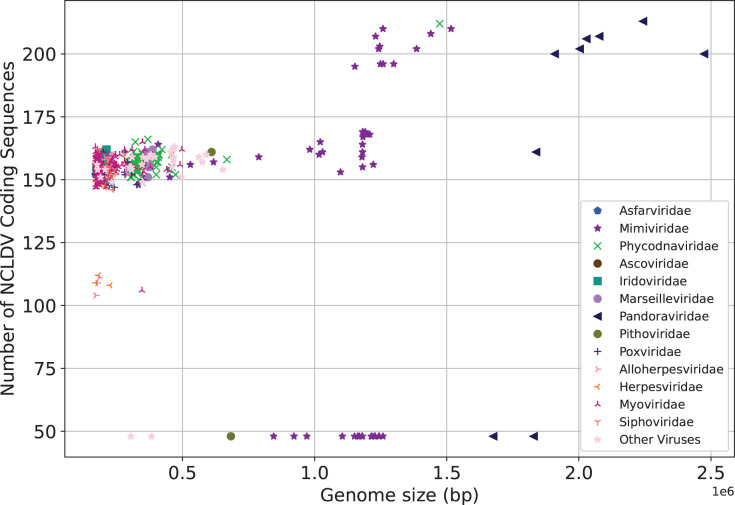
Number of coding sequences of nucleocytoplasmic large DNA viruses. Each dot represents a sequence. X-axis represents the genome size, and Y-axis represents the number of coding sequences of NCLDV. The representative families of giant viruses, Mimiviridae and Pandoraviridae have larger genome size and more coding sequences.

We next make preliminary statistics on the gene distribution to understand the giant virus. All known giant viruses belong to the phylum Nucleocytoviricota (nucleocytoplasmic large DNA viruses, NCLDVs), which contains many unique genes that cannot be found in other life forms (Ogata, Toyoda & Tomaru, 2009). Here we only consider the coding sequence. We plot the number of NCLDV coding sequences on the y-axis, and the genome size on the x-axis. The results are shown in Fig. 3. The representative families of giant viruses, Mimiviridae and Pandoraviridae have larger genome size and more coding sequences (Mimiviridae: purple star, Pandoraviridae: black triangle). In addition, the G+C content of Pandoraviridae is the highest among NCLDVs (average 61.3%), and the earliest discovered virus group, Mimiviridae has the lowest G+C content (average 26.3%, Table S1).

### Nearest neighbor classification of the genomes of giant viruses

The similarity of genomic distribution between giant viruses and bacteria, archaea, other viruses is of great importance to understand their phylogenetic relationship. We use a new 18-dimensional natural vector with covariance to measure it. There are 24,250 different biological sequences in these four datasets (Genomes of giant virus, bacteria, archaea, and virus), of which 677 sequences belong to 31 groups for giant virus dataset. Each sequence is converted into an 18-dimensional natural vector first. For each sequence in giant virus dataset, the closest sequence is then calculated and the sequence type is checked. The Euclidean distance between two 18-dimensional natural vectors is used to measure the biological similarity of the corresponding sequences. The nearest neighbor classification results are shown in Table 3.

**Table 3:** Statistics of the closest sequence types for giant virus dataset.

	Group name of large genome virus	Seq Nu.	Nearest type: Bacteria	Nearest type: Virus	SeqLen			
					Max	Min	Mean	Median
1	Polydnaviridae	230	1	229	41,573	1,533	6,684	3,836
2	Myoviridae	138	67	71	4,97,513	170,286	229,450	191,652
3	Phycodnaviridae	82	76	6	1,473,573	171,045	362,340	329,946
4	Mimiviridae	59	59	--	1,516,267	317,278	1,048,973	1,181,042
5	Poxviridae	39	34	5	359,853	170,560	244,824	224,499
6	Alloherpesviridae	20	17	3	295,146	171,096	224,435	223,830
7	Herpesviridae	17	12	5	233,501	171,823	203,228	204,237
8	Marseilleviridae	13	13	--	386,631	360,610	370,058	369,360
9	Pandoraviridae	12	12	--	2,473,870	1,676,110	2,058,604	2,015,816
10	Iridoviridae	10	10	--	220,222	186,250	202,184	201,674
11	Siphoviridae	10	10	--	279,967	185,683	223,445	219,073
12	Faustovirus	8	8	--	470,659	455,803	464,660	465,984
13	Nimaviridae	7	7	--	309,286	300,223	305,918	305,119
14	Cedratvirus	4	4	--	589,068	560,887	578,546	582,115
15	Pithoviridae	3	3	--	683,254	610,033	634,440	610,033
16	*Apis mellifera* filamentous virus	2	2	--	496,396	496,396	496,396	496,396
17	Ascoviridae	2	--	2	186,262	174,059	180,161	180,161
18	Kaumoebavirus	2	2	--	350,731	350,731	350,731	350,731
19	Lausannevirus	2	2	--	346,754	346,754	346,754	346,754
20	Mollivirus	2	2	--	651,523	651,523	651,523	651,523
21	Pacmanvirus	2	2	--	395,405	395,405	395,405	395,405
22	Bamfordvirae	2	2	--	380,011	349,275	364,643	364,643
23	Baculoviridae	2	--	2	178,733	176,677	177,705	177,705
24	Asfarviridae	1	--	1	170,101	170,101	170,101	170,101
25	Brazilian cedratvirus	1	1	--	460,038	460,038	460,038	460,038
26	Brazilian Marseilleviridae	2	2	--	362,276	362,276	362,276	362,276
27	Cannes 8 virus	1	1	--	374,041	374,041	374,041	374,041
28	Glossinavirus	1	1	--	190,032	190,032	190,032	190,032
29	Nudiviridae	1	1	--	231,621	231,621	231,621	231,621
30	Insectomime	1	1	--	382,785	382,785	382,785	382,785
31	Malacoherpesviridae	1	1	--	207,439	207,439	207,439	207,439

Except for Polydnaviridae and Myoviridae, the nucleotide distribution of most sequences is similar to that of bacteria $\left({{285} \over {309}} \times 100\% = 92.23\%\right)$. About half of the 138 Myoviridae sequences are close to the bacterial genomes (67 sequences) and half to the viral genomes (71 sequences). One of the reasons for this phenomenon is that the sequence length of Myoviridae is similar to that of some bacteria. Of the 230 Polydnaviridae sequences, 229 are closest to the virus sequence. These 230 sequences belong to two genera, *Ichnovirus* (IV) and *Bracovirus* (BV), and five species (Table S2). *Glypta fumiferanae ichnovirus* (GfIV) has 105 segments, which is the virus with the most segments among all segmented viruses. The segment length is about 2,777 bp, which is shorter than bacterial sequence. In addition, all sequences of three representative giant virus families, Mimiviridae, Marseilleviridae, and Pandoraviridae are closer to bacterial sequences.

### Phylogenetic analysis of the genomes of giant viruses

**Figure 4:**
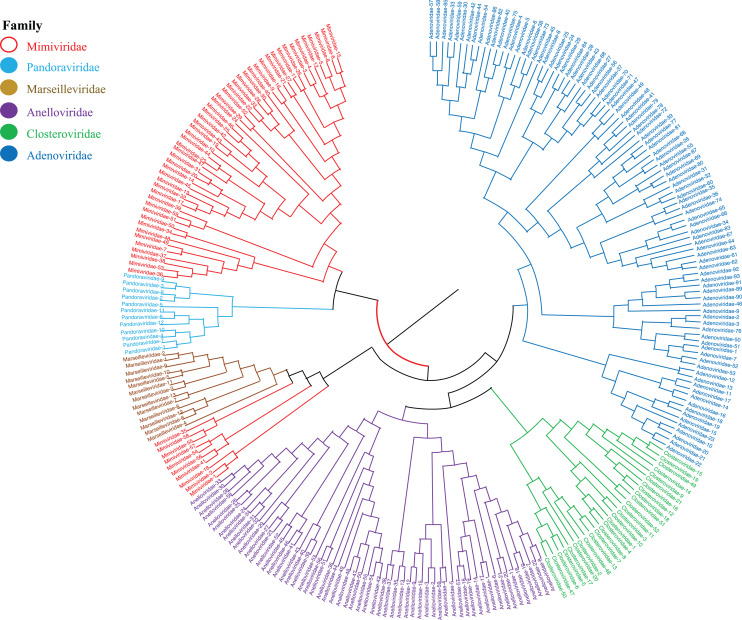
Phylogenetic tree for the three representative families (Mimiviridae, Pandoraviridae, and Marseilleviridae) of giant virus and three other viral families (Adenoviridae, Anelloviridae, and Closteroviridae). Different colors represent different families.

**Figure 5:**
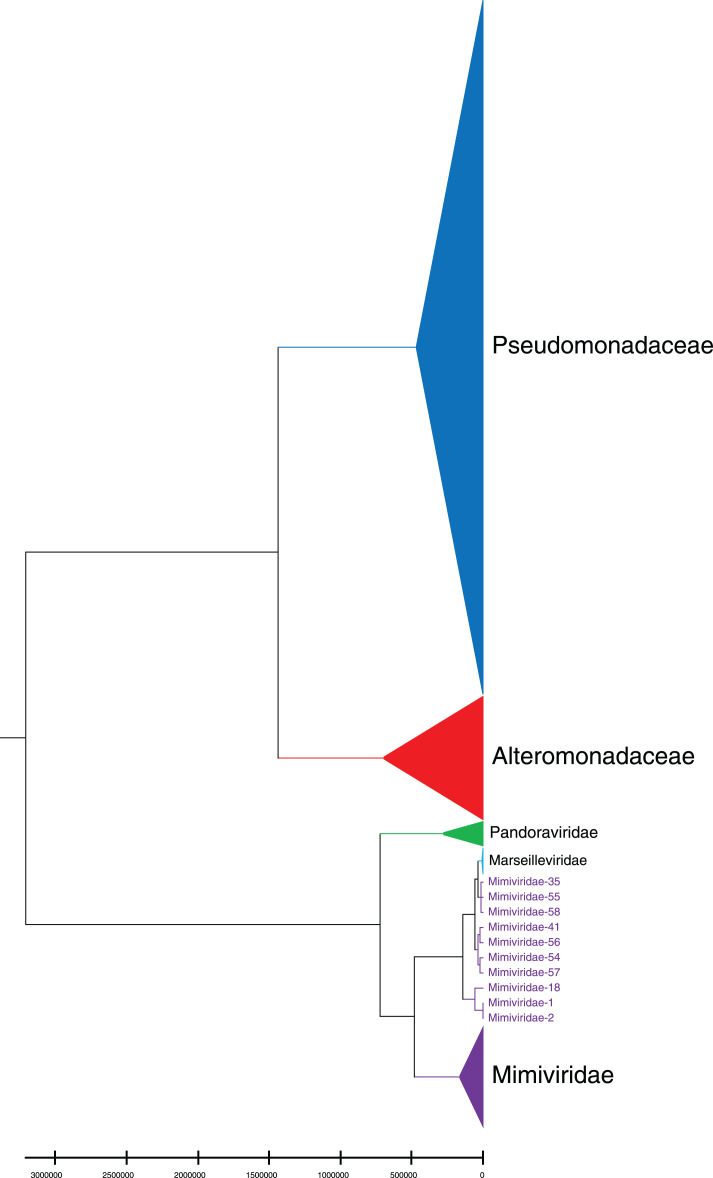
Phylogenetic tree for the three representative families (Mimiviridae, Pandoraviridae, and Marseilleviridae) of giant virus and two bacterial families (Pseudomonadaceae and Alteromonadaceae). Different colors represent different families.

**Figure 6:**
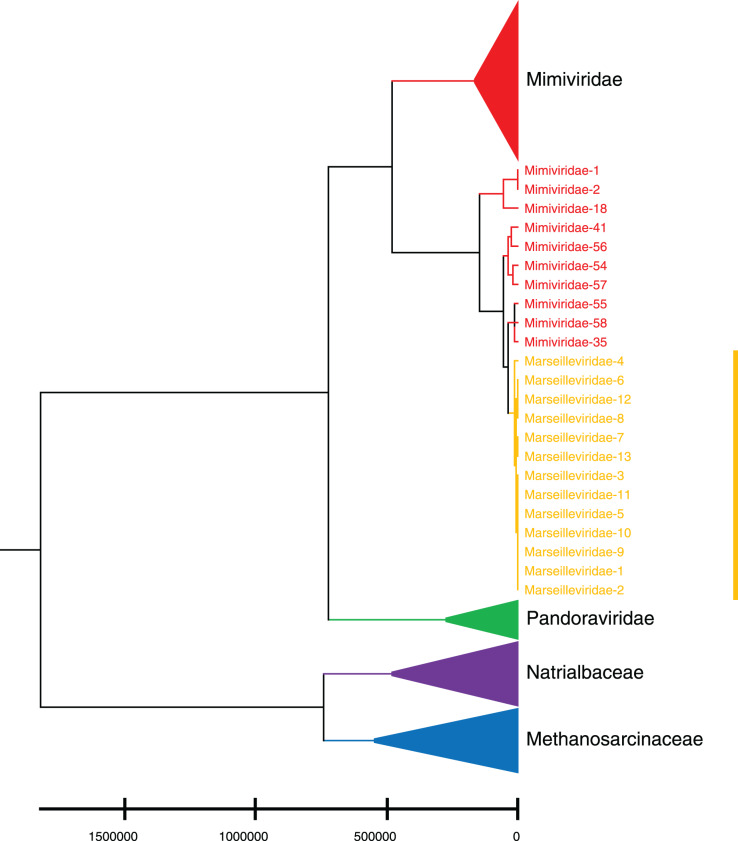
Phylogenetic tree for the three representative families (Mimiviridae, Pandoraviridae, and Marseilleviridae) of giant virus and two archaea families (Methanosarcinaceae and Natrialbaceae). Different colors represent different families.

Phylogenetic relationships between giant virus and bacteria, archaea, other viruses are studied using our new alignment-free method. We select three representative families from giant virus (Mimiviridae, Pandoraviridae, and Marseilleviridae) and randomly select two or three families from other three datasets to construct the phylogenetic tree. Figure 4 shows the phylogenetic tree of three other virus families (Adenoviridae, Anelloviridae, and Closteroviridae) and the three families of giant virus, different groups are marked in different colors. All groups are separate except Mimiviridae. To clearly observe the relationships between the three giant virus families, two bacterial families, Pseudomonadaceae and Alteromonadaceae are selected for further analysis. The phylogenetic result in Fig. 5 shows that all sequences from the same family are clustered except Mimiviridae. Beyond that, two archaea families, Methanosarcinaceae and Natrialbaceae are selected to construct the phylogenetic tree, the same family groups together except Mimiviridae is shown in Fig. 6. The algorithm of constructing the phylogenetic tree is unweighted pair-group method with arithmetic mean (UPGMA), which is an approach of constructing rooted tree based on distance matrix. The distance matrix is calculated using the commonly used Euclidean distance. In Figs. 4–6, the same 10 Mimiviridae species and Marseilleviridae species are clustered together. The sequence information is displayed in Table S3. The length of the 10 sequences is shorter than that of other viruses of Mimiviridae, but similar to some viruses of Marseilleviridae. The average sequence length of Marseilleviridae is about 370 kbp, that of Mimiviridae is about 1,000 kbp, and the length of 10 sequences is hundreds of thousands of bp.

To demonstrate the importance of natural vector with six-dimensional covariance component in characterizing biological sequence, we compare it with the traditional 12-dimensional natural vector and another alignment-free method, position natural vector (He et al., 2020) on the same three small datasets. The phylogenetic trees based on 12-dimensional natural vector are shown in Figs. S1 to S3. In Fig. S1, the sequences of Adenoviridae colored navy blue are not clustered together. In Fig. S2, there are two extra sequences from Mimiviridae clustered with Marseilleviridae. In Fig. S3, the sequences of Methanosarcinaceae are not grouped together. Additionally, the results of position natural vector method are shown in Figs. S4 to S6. In Fig. S4, a sequence from Marseilleviridae is incorrectly clustered with Mimiviridae. Figure S5 shows an unstable result compared with Fig. 5. In Fig. S6, the sequences of three families Methanosarcinaceae, Pandoraviridae, and Natrialbaceae are mixed. In conclusion, the tree based on our new 18-dimensional natural vector gives more reasonable and interpretable results.

Haursdorff distance can reflect the genetic distance between groups, and we want to analyze the interspecific differences of the above 10 families. Mathematically, Haursdorff distance is used to calculate the distance between point sets. If X and Y are two non-empty point sets, then the Haursdorff distance is defined as:

(9) $$\matrix{ {d( {X,Y} ) = \max \left\{ {\mathop {\sup }\limits_{x \;\in\; X} \mathop {\;\inf }\limits_{y \;\in\; Y} d( {x,y} ),\mathop {\sup }\limits_{y \;\in\; Y} \mathop {\;\inf }\limits_{x \;\in\; X} d( {x,y} )} \right\}.} \cr }$$

The distance calculation can be implemented through the code of the MathWorks community: https://ww2.mathworks.cn/matlabcentral/fileexchange/26738-hausdorff-distance. Each sequence is transformed into an 18-dimensional natural vector, then Haursdorff distance between groups is calculated, and UPGMA algorithm based on distance matrix is used to construct the tree, as shown in Fig. S7. Mimiviridae is marked with circles, and Marsellieviridae with triangles. Mimiviridae is closer to the root of the tree than Marsellieviridae.

### Time and phylogeny comparison with previous covariance method

The previous study has shown that nucleotide covariance is related to the phylogenetics of fungi (Zhao, Tian & Yau, 2018), we redefine the nucleotide covariance to make it simpler and more general.

The previous covariance definition is:

(10) $${Cov( {k,l} ) = {{Cov( {A,B} )} \over N},} $$

$k,l \;\in\; \left\{ {A,C,G,{\rm{T\;or\;U}}} \right\}.\;A = \left\{ {{a_1},{a_2}, \ldots ,{a_n}} \right\},B = \left\{ {{b_1},{b_2}, \ldots ,{b_m}} \right\}$ are the position of k and l, respectively.

• If $m = n$, $\displaystyle Cov( {A,B} ) = \sum\nolimits_{i = 1}^n {{{( {{a_i} - \sum\nolimits_{i = 1}^n {{{{a_i}} \over n}} } )( {{b_i} - \sum\nolimits_{i = 1}^m {{{{b_i}} \over m}} } )} \over n}}$.
• If $m \ne n$ (assume $m \gt n$), the covariance between A and any n values in B is computed and the average of these $C_m^n$ results is $Cov( {A,B} )$.

For a bacterial sequence (Accession number in Genbank is AP018515, sequence length is 3,041,114 bp), the number of nucleotide T is m = 723,764 and the number of nucleotide C is n = 799,865, the covariance of T and C using the previous definition is:

(11) $${Cov( {T,C} ) = {{Cov( {A,B} )} \over {3041114}},} $$

Suppose the positions of T and C are $A = \left\{ {{a_1},{a_2}, \ldots ,{a_{723764}}} \right\},B = \left\{ {{b_1},{b_2}, \ldots ,{b_{799865}}} \right\}$, respectively, then the covariance between A and any 723,764 values in B is computed and the average of these $C_{799865}^{723764}$ results is:

(12) $$\eqalign{ Cov( {A,B} ) =& {1 \over {C_{799865}^{723764}}}{\sum}_{\left\{{j_1},\ {j_2},\;\ldots ,{j_{723764}}\right\} \;\in\; B\ =\; \left\{ {b_1},\;{b_2},\; \ldots ,{b_{799865}} \right\}} \cr& \sum\limits_{i = 1}^{723764} {{{( {{a_i} - \sum\nolimits_{i = 1}^{723764} {{{{a_i}} \over n}} } )( {{b_i} - \sum\nolimits_{i = 1}^{723764} {{{{b_{{j_i}}}} \over m}} } )} \over {723764}}},}$$

If the number of two nucleotides is quite different, the above formula (12) is too complicated and difficult to compute, which will take a lot of time to calculate. Our new nucleotide covariance definition is $Cov( {k,l} ) = \sum\nolimits_{i = 1}^N {{{\left[ {i - {\mu _k}} \right]\left[ {i - {\mu _l}} \right]{w_{kl}}( {{s_i}} )} \over {N\sqrt {{n_k}} \sqrt {{n_l}} }}}$, there is no need to compute $C_{799865}^{723764}$ values, so the calculation of covariance takes less time.

**Figure 7:**
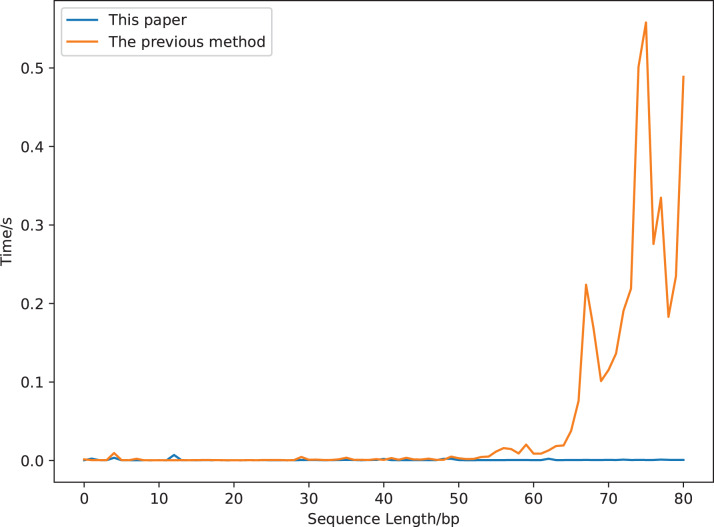
The calculation time comparison of 18-dimensional natural vectors under the covariance definitions of this article and the previous study.

We compare the running time of the two definitions using a sequence segment with a length of 90 bp (gaggaagtaaaagtcgtaacaaggtttccttccgggtgtagcacctgccgaagcctcccgcagcgactctaaagaaactgcgcagtctgc, a segment of a sequence whose accession number is DQ525472). The results are displayed in Fig. 7. The calculation time increases greatly with the increase of the sequence length for the previous covariance method, but is robust for our new method. It costs about 0.5 s to compute the previous covariance vector of a sequence with length 75 bp, which is much longer than that of our method (Fig. 7). While it only costs 0.017 s to compute our covariance vector of a sequence with length 813 bp (GenBank accession number is DQ525472).

The previous covariance method is tested on the fungi DNA barcode dataset, so we also apply our method on the same dataset. To show that our method can also be used for phylogenetic analysis, we choose 311 nucleotide sequences of DNA barcode from nine species of fungi (*Monosporascus cannonballus, Peniophorella praetermissa, Biatora helvola, Ceratobasidium cereale, Rhodotorula glutinis, Geosmithia landonii, Fusarium cf. solani, Acarospora smaragdula, Acaulospors kentinensis*) to construct phylogenetic tree, as shown in Fig. S8. The nine groups can be separated based on our method, while a sequence from *Peniophorella praetermissa* doesn’t cluster together with *Peniophorella praetermissa* based on the previous covariance method.

## Discussion

As a phylogenetic researcher with mathematical background, we propose a new alignment-free method to compare sequences from the perspective of statistics, which overcomes the shortcomings of high demand for computer experimental setup of the traditional alignment method. The new 18-dimensional natural vector method improves the 12-dimensional natural vector, and successfully numerically characterizes a large number of microbial genome data. In this way, each sequence corresponds to a point in 18-dimensional Euclidean space. The sequence similarity can be compared quickly and accurately. The traditional 12-dimensional natural vector only considers the distribution of a single nucleotide, but ignores the relationship between nucleotides. The new 18-dimensional natural vector method takes all these features into account, and contains the counts, average position and central moment of single nucleotide as well as the covariance between nucleotides. The rationality of the method is verified by testing on five datasets, archaea, bacteria, virus, fungi (genome), fungi (DNA barcode) using the convex hull classification. The results show that the six-dimensional covariance vector is a necessary condition to characterize the sequence. A further verification is performed on a special dataset, giant virus. The results show that the new 18-dimensional vector plays an important role in classification and evolution.

Many details of our article are worth discussing. First, the previous study has shown that the covariance between nucleotides is related to the phylogeny of fungi (Zhao, Tian & Yau, 2018). However, the limitation of the old covariance definition makes it difficult to calculate in a reasonable time if the number of two nucleotides in a sequence is quite different. Therefore, we propose a new and more natural covariance definition, which can be computed quickly. The new proposed method has obvious advantages in processing a large number of sequences and detecting the similarity and difference between sequences. Second, our proposed method has important practical significance in sequence alignment. For some sequence similarity search tools (For example, BLAST, MUSCLE), searching and aligning sequences will be very time-consuming (Figs. S9 and S10). The new method overcomes these shortcomings. It can not only naturally and effectively describe the distribution of four nucleotides, but also need less memory to store the 18-dimensional numerical vectors. Our method is promising for studying sequence alignment problems with large-scale data. Third, the microbial nucleotide sequences are utilized to verify the rationality of our new covariance definition. The data is abundant and easy to process, which is convenient for other researchers to repeat the experiment. The method can also be used for sequence comparison of other organisms, such as plants, vertebrates and invertebrates. It is beyond the scope of this article, we will study it in future work. Fourth, we use the convex hull classification results to show the necessity of six-dimensional covariance vectors in characterizing sequence. The results are reliable because the convex hull principle has strict theoretical supports: optimization background (Zhao, Tian & Yau, 2018), protein classification (Zhao et al., 2019) and the geometry construction of virus genome space (Sun et al., 2021). Fifth, we only use 18-dimensional natural vector to study the relationship of giant virus and bacteria, archaea, other viruses. Further studies can also be explored, for example, the geometric construction of genome space, evolution analysis. Sixth, the covariance definition of nucleotide can be extended to protein sequences. There are 20 amino acids: A, R, N, D, C, Q, E, G, H, I, L, K, M, F, P, S, T, W, Y, V. The natural vector with the covariance component of a protein sequence consists of four kinds of features including the counts ( ${n_k}$), the average positions ( ${\mu _k}$) and the central moment of position ( $D_2^k$) of the 20 amino acids as well as the covariance between different amino acids ( $Cov( {k,l} )$, k or l $\;\in\; \left\{ {{\rm{A}},{\rm{R}},{\rm{N}},{\rm{D}},{\rm{C}},{\rm{Q}},{\rm{E}},{\rm{G}},{\rm{H}},{\rm{I}},{\rm{L}},{\rm{K}},{\rm{M}},{\rm{F}},{\rm{P}},{\rm{S}},{\rm{T}},{\rm{W}},{\rm{Y}},{\rm{V}}} \right\}$):

${\rm{[}}{{\rm{n}}_{\rm{A}}},{{\rm{n}}_{\rm{R}}},{{\rm{n}}_{\rm{N}}},{{\rm{n}}_{\rm{D}}},{{\rm{n}}_{\rm{C}}},{{\rm{n}}_{\rm{Q}}},{{\rm{n}}_{\rm{E}}},{{\rm{n}}_{\rm{G}}},{{\rm{n}}_{\rm{H}}},{{\rm{n}}_{\rm{I}}},{{\rm{n}}_{\rm{L}}},{{\rm{n}}_{\rm{K}}},{{\rm{n}}_{\rm{M}}},{{\rm{n}}_{\rm{F}}},{{\rm{n}}_{\rm{P}}},{{\rm{n}}_{\rm{S}}},{{\rm{n}}_{\rm{T}}},{{\rm{n}}_{\rm{W}}},{{\rm{n}}_{\rm{Y}}},{{\rm{n}}_{\rm{V}}},$

$\rm {\rm \mu _{\rm{A}}},{\mu _{\rm{R}}},{\mu _{\rm{N}}},{\mu _{\rm{D}}},{\mu _{\rm{C}}},{\mu _{\rm{Q}}},{\mu _{\rm{E}}},{\mu _{\rm{G}}},{\mu _{\rm{H}}},{\mu _{\rm{I}}},{\mu _{\rm{L}}},{\mu _{\rm{K}}},{\mu _{\rm{M}}},{\mu _{\rm{F}}},{\mu _{\rm{P}}},{\mu _{\rm{S}}},{\mu _{\rm{T}}},{\mu _{\rm{W}}},{\mu _{\rm{Y}}},{\mu _{\rm{V}}},$

${\rm{D}}_2^{\rm{A}},{\rm{D}}_2^{\rm{R}},{\rm{D}}_2^{\rm{N}},{\rm{D}}_2^{\rm{D}},{\rm{D}}_2^{\rm{C}},{\rm{D}}_2^{\rm{Q}},{\rm{D}}_2^{\rm{E}},{\rm{D}}_2^{\rm{G}},{\rm{D}}_2^{\rm{H}},{\rm{D}}_2^{\rm{I}},{\rm{D}}_2^{\rm{L}},{\rm{D}}_2^{\rm{K}},{\rm{D}}_2^{\rm{M}},{\rm{D}}_2^{\rm{F}},{\rm{D}}_2^{\rm{P}},{\rm{D}}_2^{\rm{S}},{\rm{D}}_2^{\rm{T}},{\rm{D}}_2^{\rm{W}},{\rm{D}}_2^{\rm{Y}},{\rm{D}}_2^{\rm{V}},$

${\rm{Cov}}( {{\rm{A}},{\rm{R}}} ),\;{\rm{Cov}}( {{\rm{A}},{\rm{N}}} ),\;{\rm{Cov}}( {{\rm{A}},{\rm{D}}} ),\;{\rm{Cov}}( {{\rm{A}},{\rm{C}}} ),\;{\rm{Cov}} ( {{\rm{A}},{\rm{Q}}} ),\;{\rm{Cov}}( {{\rm{A}},{\rm{E}}} ), {\rm{Cov}}( {{\rm{A}},{\rm{G}}} ),  {\rm{Cov}} ( {{\rm{A}},{\rm{H}}} ),\;{\rm{Cov}}( {{\rm{A}},{\rm{I}}} ),\;{\rm{Cov}}( {{\rm{A}},{\rm{L}}} ),$

${\rm{Cov}}( {{\rm{A}},{\rm{K}}} ),\;{\rm{Cov}}( {{\rm{A}},{\rm{M}}} ),\;{\rm{Cov}}( {{\rm{A}},{\rm{F}}} ),\;{\rm{Cov}}( {{\rm{A}},{\rm{P}}} ),  {\rm{Cov}}( {{\rm{A}},{\rm{S}}} ),\;{\rm{Cov}}( {{\rm{A}},{\rm{T}}} ),\;{\rm{Cov}}( {{\rm{A}},{\rm{W}}} ),  {\rm{Cov}}( {{\rm{A}},{\rm{Y}}} ),\;{\rm{Cov}}( {{\rm{A}},{\rm{V}}} ), \ldots {\rm{Cov}}( {{\rm{Y}},{\rm{V}}} ){\rm]}. $

So, the natural vector for a protein sequence is 250-dimensional, of which the covariance component is 190-dimensional. Seventh, we emphasize that dealing with the massive data sets and viewing biological problems from a mathematical perspective will lead to a deeper and more rapid understanding of their nature than relying solely on expensive experimentation. Because mathematicians may see some profound and unexpected structures and invent new mathematical methods to understand the high-dimensional properties and complex dynamics of biological problems (Zhao, Pei & Yau, 2020). Therefore, we propose a new mathematical numerical representation to describe the nucleotide distribution of sequences. Compared with direct (linguistic) method that avoid text conversion, for example, k-mer method, our method has two advantages: (1) It doesn’t depend on any assumption because there is no need to determine k. For a genome sequence, k-mer is a sequence segment of length k (Dai, Yang & Wang, 2008; Leimeister & Morgenstern, 2014). For each given k, the number of k-mer is fixed: 1-mers indicate A, C, G, T. Our method only considers the distribution of 1-mers; (2) It is more statistically significant than k-mers because we consider three features of 1-mers, even we can extend the definition by combining 18-dimensional vector and k-mers: k-mer natural vector. The distribution of 2-mers (2-mers include AA, AC, AG, AT, CA, CC, CG, CT, GA, GC, GG, GT, TA, TC, TG, TT) can be described as:

${\rm[}{{\rm{n}}_{{\rm{AA}}}},{{\rm{n}}_{{\rm{AC}}}},{n_{AG}},{n_{AT}},{n_{CA}},{n_{CC}},{n_{CG}},{n_{CT}},{n_{{\rm{GA}}}},{{\rm{n}}_{{\rm{GC}}}},{{\rm{n}}_{{\rm{GG}}}},{{\rm{n}}_{{\rm{GT}}}},{{\rm{n}}_{{\rm{TA}}}},{{\rm{n}}_{{\rm{TC}}}},{{\rm{n}}_{{\rm{TG}}}},{{\rm{n}}_{{\rm{TT}}}},$

$\rm {\mu _{{\rm{AA}}}},{\mu _{{\rm{AC}}}},{\mu _{{\rm{AG}}}},{\mu _{{\rm{AT}}}},{\mu _{{\rm{CA}}}},{\mu _{{\rm{CC}}}},{\mu _{{\rm{CG}}}},{\mu _{{\rm{CT}}}},{\mu _{{\rm{GA}}}},{\mu _{{\rm{GC}}}},{\mu _{{\rm{GG}}}},{\mu _{{\rm{GT}}}},{\mu _{{\rm{TA}}}},{\mu _{{\rm{TC}}}},{\mu _{{\rm{TG}}}},{\mu _{{\rm{TT}}}},$

${\rm{D}}_2^{{\rm{AA}}},{\rm{D}}_2^{{\rm{AC}}},{\rm{D}}_2^{{\rm{AG}}},{\rm{D}}_2^{{\rm{AT}}},{\rm{D}}_2^{{\rm{CA}}},{\rm{D}}_2^{{\rm{CC}}},{\rm{D}}_2^{{\rm{CG}}},{\rm{D}}_2^{{\rm{CT}}},{\rm{D}}_2^{{\rm{GA}}},{\rm{D}}_2^{{\rm{GC}}},{\rm{D}}_2^{{\rm{GG}}},{\rm{D}}_2^{{\rm{GT}}},{\rm{D}}_2^{{\rm{TA}}},{\rm{D}}_2^{{\rm{TC}}},{\rm{D}}_2^{{\rm{TG}}},{\rm{D}}_2^{{\rm{TT}}},$

${\rm{Cov}}( {{\rm{AA}},{\rm{AC}}} ),{\rm{Cov}}( {{\rm{AA}},{\rm{AG}}} ),\;{\rm{Cov}}( {{\rm{AA}},{\rm{AT}}} ),{\rm{Cov}}( {{\rm{AA}},{\rm{CA}}} ), {\rm{Cov}}( {{\rm{AA}},{\rm{CC}}} ), \ldots ,  {\rm{Cov}}( {{\rm{TG}},{\rm{TT}}\;} ){\rm]}.$

Therefore, our method gives a new mathematical framework to describe the distributions of k-mers, and it may be of great significance for the application of efficient and large-scale sequence alignment in the future.

## Conclusions

In this article, a new six-dimensional covariance vector is proposed to reflect the correlation between nucleotides, which improves the traditional 12-dimensional natural vector. The new 18-dimensional natural vector is tested on six datasets, including five genome sequence datasets (archaea, bacteria, virus, fungi and giant virus) and one gene sequence dataset (fungi). First of all, we perform the convex hull classification. The results show that the classification performance of 18-dimensional natural vector is better than that of 12-dimensional vector, which verifies the necessity of 6-dimensional covariance vector in characterizing biological sequences. Furthermore, we study a special virus, giant virus. Statistical analysis gives us a preliminary understanding of the representative families of giant virus, Mimiviridae, Pandoraviridae and Marsellieviridae: Pandoravirus has the largest genome size and the most of the G+C content, Mimiviridae and Pandoraviridae have more coding sequences than other families. Then we analyze the relationship between giant viruses and bacteria, archaea, other viruses. Except for Polydnaviridae and Myoviridae, the nucleotide distribution of most sequences is similar to that of bacteria. And the phylogenetic trees show a stable result, that is, 10 sequences of Mimiviridae cluster with Marseilleviridae, and Mimiviridae is closer to the root of the tree than Marsellieviridae. While another alignment-free method, position natural vector, and 12-dimensional natural vector applied to the same dataset do not show stable results. Finally, we compare the time and phylogeny with the previous covariance definition. Our method needs less computation and memory, but gets accurate results. Our 18-dimensional natural vector is a powerful alignment-free method to characterize biological sequences.

## Supplemental Information

10.7717/peerj.13544/supp-1 Supplemental Information 1 The accession numbers of genomic sequences of archaea.

10.7717/peerj.13544/supp-2 Supplemental Information 2 The accession numbers of genomic sequences of bacteria.

10.7717/peerj.13544/supp-3 Supplemental Information 3 The accession numbers of genomic sequences of virus.

10.7717/peerj.13544/supp-4 Supplemental Information 4 The accession numbers of genomic sequences of fungi.

10.7717/peerj.13544/supp-5 Supplemental Information 5 The accession numbers of genomic sequences of giant virus.

10.7717/peerj.13544/supp-6 Supplemental Information 6 The accession numbers of DNA barcode of fungi.

10.7717/peerj.13544/supp-7 Supplemental Information 7 Phylogenetic tree for the three representative families of giant viruses (Mimiviridae, Pandoraviridae, and Marseilleviridae) and three other viral families (Adenoviridae, Anelloviridae, and Closteroviridae) based on 12-dimensional natural vector.

10.7717/peerj.13544/supp-8 Supplemental Information 8 Phylogenetic tree for the three representative families of giant viruses (Mimiviridae, Pandoraviridae, and Marseilleviridae) and two bacterial families (Pseudomonadaceae and Alteromonadaceae) based on 12-dimensional natural vector.

10.7717/peerj.13544/supp-9 Supplemental Information 9 Comparison between BLAST and our method. We select a viral genome sequence (Accession number in GenBank is NC_035619, sequence length is 30620 bp) and search its similar sequences.
(A) The parameters choice for the similar sequence search using BLAST (https://blast.ncbi.nlm.nih.gov/Blast.cgi). (B) Similar sequence search results using BLAST for the viral genome sequence (Accession number in GenBank is NC_035619). (C) The top ten similar sequences using our 18-dimensional natural vector method. Here the biological distance between two sequences is measured using the Euclidean distance of their corresponding 18-dimensional natural vectors, which is commonly used in our previous studies.
It takes about 20 min for BLAST to get three similar sequences (Accession number are NC_002685.2, NC_020074.1, and NC_004037.2) for the target sequence, and the percentage identities are 73.05%, 72.75%, and 73.76%, respectively (Figure S9B). Using our 18-dimensional natural vector method, it takes about 0.024914 s to get the similar sequences from our extracted Virus dataset (Table 2 in the main text). The top three similar sequences are the same using BLAST and our method, but our method runs faster. In addition, there is a sequence length limitation for BLAST, so it is inconvenient to search similar sequences for the bacterial genomic sequence. But our methods can deal with these sequences efficiently.

10.7717/peerj.13544/supp-10 Supplemental Information 10 Comparison between MUSCLE and our method. Fifteen DNA barcode sequences of fungi are selected to construct the phylogenetic tree.
(A) The phylogenetic tree using MUSCLE method (https://www.ebi.ac.uk/Tools/msa/muscle/). (B) The phylogenetic tree using our method. The algorithm to construct the phylogenetic tree is neighbor-joining (NJ), which is an approach based on distance to construct un-rooted tree. The distance matrix is calculated using the commonly used Euclidean distance.
Using our method, the two families, Glomerellaceae and Strophariaceae can be separated better. The reason is that our method is based on the distribution of nucleotides from the perspective of statistic. Furthermore, since there are many bacterial genomic sequences in our study, MUSCLE can’t get the phylogenetic results in a reasonable time. Therefore, our method is better than MUSCLE in some sense.

10.7717/peerj.13544/supp-11 Supplemental Information 11 Phylogenetic tree for the three representative families of giant viruses (Mimiviridae, Pandoraviridae, and Marseilleviridae) and two archaea families (Methanosarcinaceae and Natrialbaceae) based on 12-dimensional natural vector.

10.7717/peerj.13544/supp-12 Supplemental Information 12 Phylogenetic tree for the three representative families of giant viruses (Mimiviridae, Pandoraviridae, and Marseilleviridae) and three other viral families (Adenoviridae, Anelloviridae, and Closteroviridae) based on 18-dimensional position natural vector.

10.7717/peerj.13544/supp-13 Supplemental Information 13 Phylogenetic tree for the three representative families of giant viruses (Mimiviridae, Pandoraviridae, and Marseilleviridae) and two bacterial families (Pseudomonadaceae and Alteromonadaceae) based on 18-dimensional position natural vector.

10.7717/peerj.13544/supp-14 Supplemental Information 14 Phylogenetic tree for the three representative families of giant viruses (Mimiviridae, Pandoraviridae, and Marseilleviridae) and two archaea families (Methanosarcinaceae and Natrialbaceae) based on 18-dimensional position natural vector.

10.7717/peerj.13544/supp-15 Supplemental Information 15 Phylogenetic tree for the viral, archaea and bacterial families based on Haursdorff distance and 18-dimensional Natural Vector algorithm. Mimiviridae is closer to the root of the tree than Marsellieviridae.

10.7717/peerj.13544/supp-16 Supplemental Information 16 Phylogenetic tree of nine species of fungi. (A) Phylogenetic tree of nine species of fungi based on our method. (B) Phylogenetic tree of nine species of fungi based on the previous correlation method.

10.7717/peerj.13544/supp-17 Supplemental Information 17 G+C contents of nucleocytoplasmic large DNA viruses.

10.7717/peerj.13544/supp-18 Supplemental Information 18 Viral sequence information of Polydnaviridae.

10.7717/peerj.13544/supp-19 Supplemental Information 19 The species information of Mimiviridae clustered with the viruses of Marseilleviridae.

10.7717/peerj.13544/supp-20 Supplemental Information 20 The supplemental MATLAB code. naturalvectordna: the function of the proposed 18-dimensional Natural Vector. intersection: the program aims to solve the linear programming problem.

## Additional Information and Declarations

### Data Availability

The following information was supplied regarding data availability: The raw data are provided in the Supplemental Files. The Archaea (ftp.ncbi.nih.gov/refseq/release/archaea), Bacteria (ftp.ncbi.nih.gov/refseq/release/bacteria) and Fungi (ftp.ncbi.nih.gov/refseq/release/fungi) datasets are available at NCBI Reference Sequence Database: https://www.ncbi.nlm.nih.gov/refseq/. The Virus Genomes dataset (ftp.ncbi.nlm.nih.gov/genomes/Viruses) is available at NCBI Genome: https://www.ncbi.nlm.nih.gov/genome.

## References

[ref-1] Almeida JS (2014). Sequence analysis by iterated maps, a review. Briefings in Bioinformatics.

[ref-2] Altschul SF, Gish W, Miller W, Myers EW, Lipman DJ (1990). Basic local alignment search tool. Journal of Molecular Biology.

[ref-3] Altschul SF, Madden TL, Schäffer AA, Zhang J, Zhang Z, Miller W, Lipman DJ (1997). Gapped BLAST and PSI-BLAST: a new generation of protein database search programs. Nucleic Acids Research.

[ref-4] Bichell R (2017). In giant virus genes, hints about their mysterious origin. https://www.npr.org/sections/health-shots/2017/04/06/522478901/in-giant-virus-genes-hints-about-their-mysterious-origin.

[ref-5] Birtles R, Rowbotham TJ, Storey C (1997). Chlamydia-like obligate parasite of free-living Amoebae. The Lancet.

[ref-6] Brandes N, Linial M (2019). Giant viruses-big surprises. Viruses.

[ref-7] Claverie J, Abergel C (2013). Open questions about giant viruses. Advances in Virus Research.

[ref-8] Conrad LS, Keith AS, Sabine H (2012). Nuclear ribosomal internal transcribed spacer (ITS) region as a universal DNA barcode marker for fungi. Proceedings of the National Academy of Sciences of the United States of America.

[ref-9] Dai Q, Yang YC, Wang TM (2008). Markov model plus k-word distributions: a synergy that produces novel statistical measures for sequence comparison. Bioinformatics.

[ref-10] Deng M, Yu C, Liang Q, He RL, Yau SST (2011). A novel method of characterizing genetic sequences: genome space with biological distance and applications. PLOS ONE.

[ref-11] Didier R, Stéphane A, Catherine R (2004). The 1.2-Megabase genome sequence of Mimivirus. Science.

[ref-12] Edgar RC (2004a). MUSCLE: multiple sequence alignment with high accuracy and high throughput. Nucleic Acids Research.

[ref-13] Edgar RC (2004b). MUSCLE: a multiple sequence alignment method with reduced time and space complexity. BMC Bioinformatics.

[ref-14] Hatje K, Kollmar M (2012). A phylogenetic analysis of the Brassicales clade based on an alignment-free sequence comparison method. Frontiers in Plant Science.

[ref-15] He L, Dong R, He RL, Yau SST (2020). Positional correlation natural vector: a novel method for genome comparison. International Journal of Molecular Sciences.

[ref-16] iBOL (2022). What is DNA barcoding?. https://ibol.org/about/dna-barcoding/.

[ref-17] Jeffrey HJ (1990). Chaos game representation of gene structure. Nucleic Acids Research.

[ref-18] Larkin MA, Blackshields G, Brown NP (2007). ClustalW and ClustalX version 2.0. Bioinformatics.

[ref-19] Legendre M, Arslan D, Abergel C (2012). Genomics of Megavirus and the elusive fourth domain of life. Communicative and Integrative Biology.

[ref-20] Leimeister C, Morgenstern B (2014). Kmacs: the k-mismatch average common substring approach to alignment-free sequence comparison. Bioinformatics.

[ref-21] Naturvetenskapliga F (2010). Current state and perspectives of fungal DNA barcoding and rapid identification procedures. Applied Microbiology and Biotechnology.

[ref-22] Ogata H, Toyoda K, Tomaru Y (2009). Remarkable sequence similarity between the dinoflagellate-infecting marine virus and the terrestrial pathogen African swine fever virus. Virology Journal.

[ref-23] Pei SJ, Dong R, Bao YM, He RL, Yau SST (2020). Classification of genomic components and prediction of genes of Begomovirus based on subsequence natural vector and support vector machine. PeerJ.

[ref-24] Philippe N, Legendre M, Doutre G, Couté Y, Poirot O, Lescot M, Arslan D, Seltzer V, Bertaux L, Bruley C, Garin J, Claverie J-M, Abergel C (2013). Pandoraviruses: amoeba viruses with genomes up to 2.5 mb reaching that of parasitic eukaryotes. Science.

[ref-25] Sun N, Pei SJ, He L, Yin CC, He RL, Yau SST (2021). Geometric construction of viral genome space and its applications. Computational and Structural Biotechnology Journal.

[ref-26] Van Etten J (2011). Giant viruses. American Scientist.

[ref-27] Vinga S (2014). Information theory applications for biological sequence analysis. Briefings in Bioinformatics.

[ref-28] Wessner DR, Dupont C, Charles T (2013). Microbiology.

[ref-29] Yin CC, Chen Y, Yau SST (2014). A measure of DNA sequence similarity by Fourier transform with applications on hierarchical clustering. Journal of Theoretical Biology.

[ref-30] Zhao RZ, Pei SJ, Yau SST (2020). New genome sequence detection via natural vector convex hull method. IEEE/ACM Transactions on Computational Biology and Bioinformatics.

[ref-31] Zhao X, Tian K, He RL, Yau SST (2019). Convex hull principle for classification and phylogeny of eukaryotic proteins. Genomic.

[ref-32] Zhao X, Tian K, Yau SST (2018). A new efficient method for analyzing fungi species using correlations between nucleotides. BMC Evolutionary Biology.

