# Supplementary figures and images for: An efficient numerical representation of genome sequence: natural vector with covariance component
Source: PeerJ. 2022 Jun 16;10:e13544. doi: 10.7717/peerj.13544 (PMC9206847; doi:10.7717/peerj.13544)

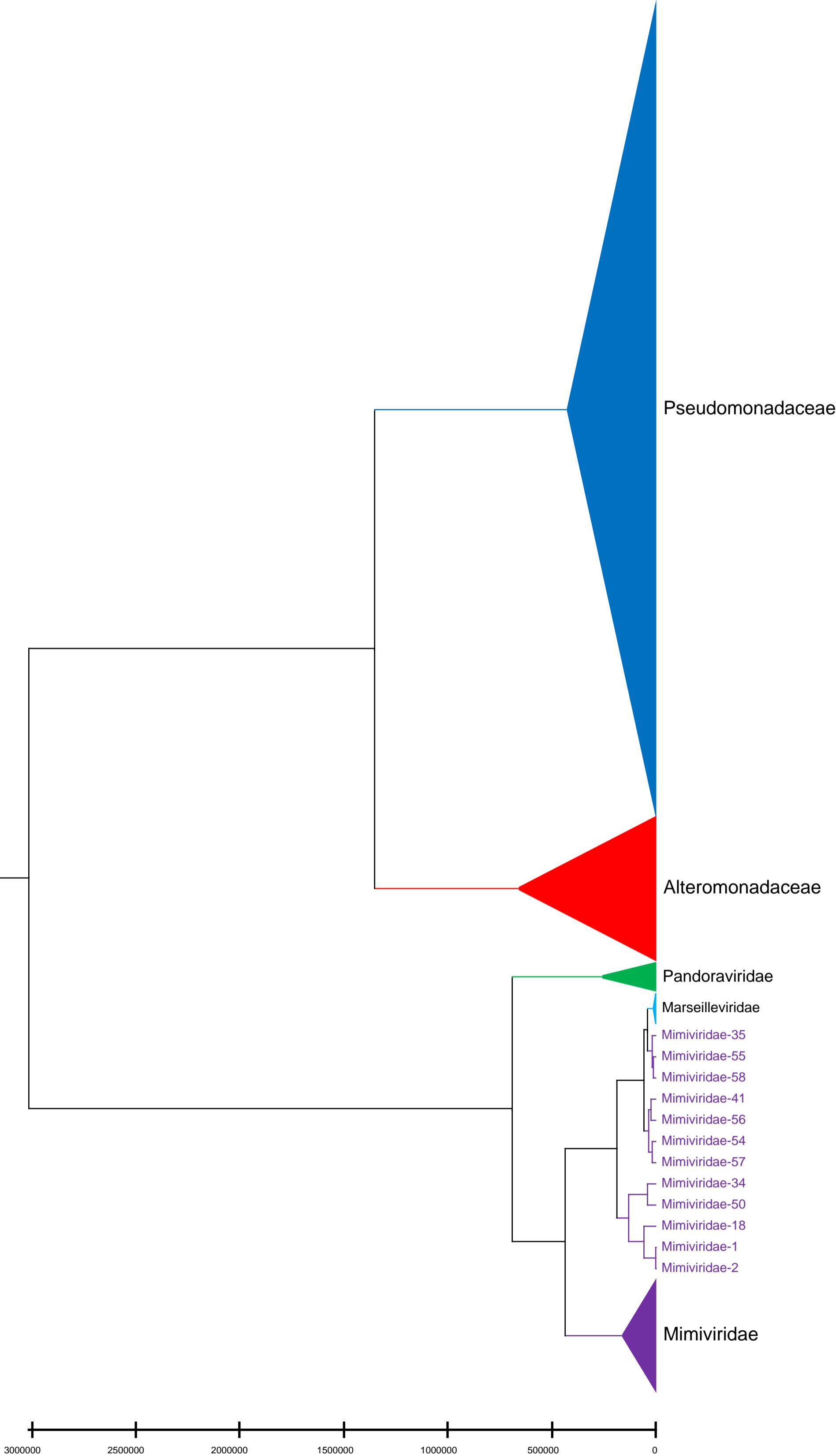

Supplement: Supplemental Information 8 [file peerj-10-13544-s008.pdf]

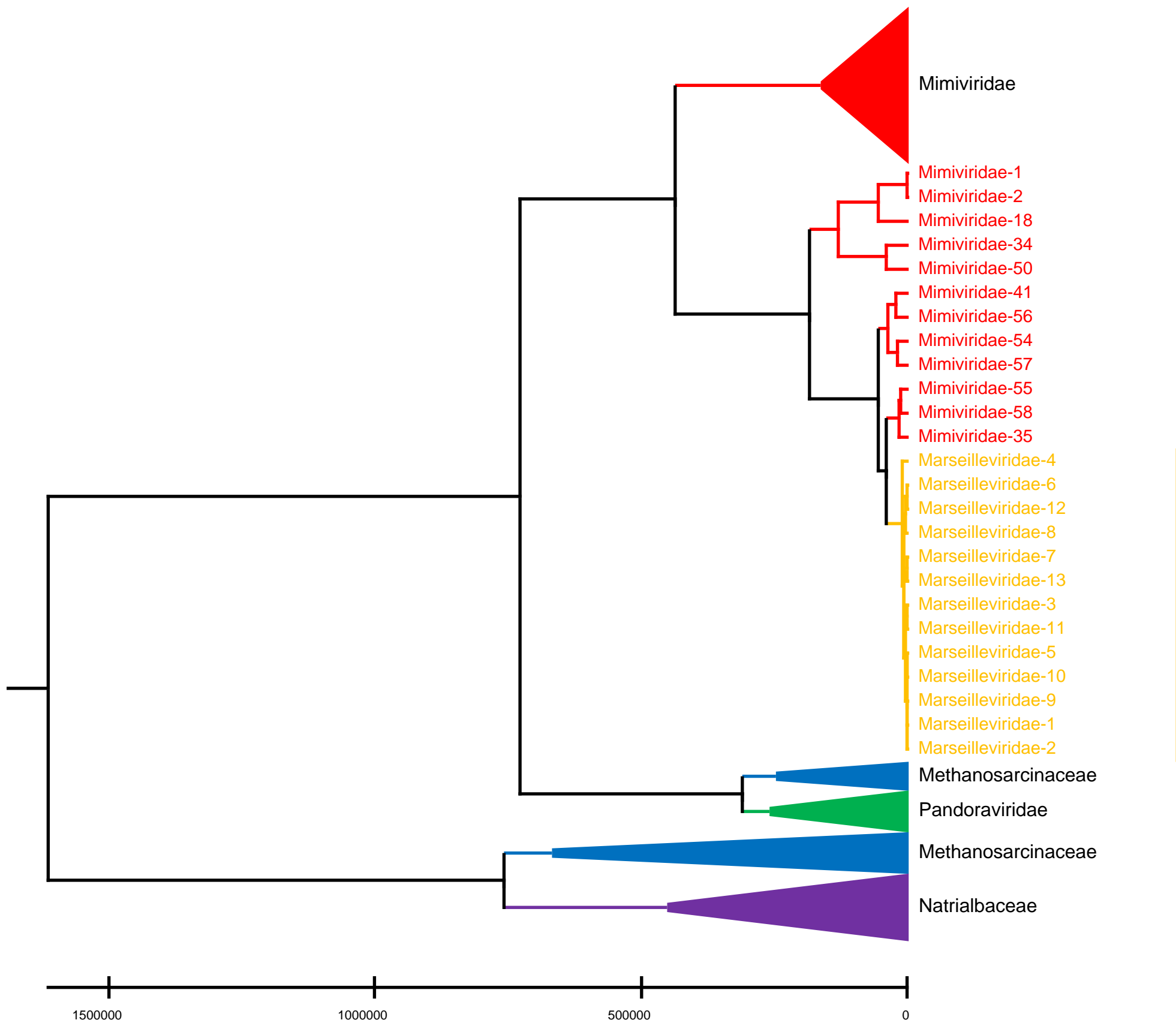

Supplement: Supplemental Information 11 [file peerj-10-13544-s011.pdf]

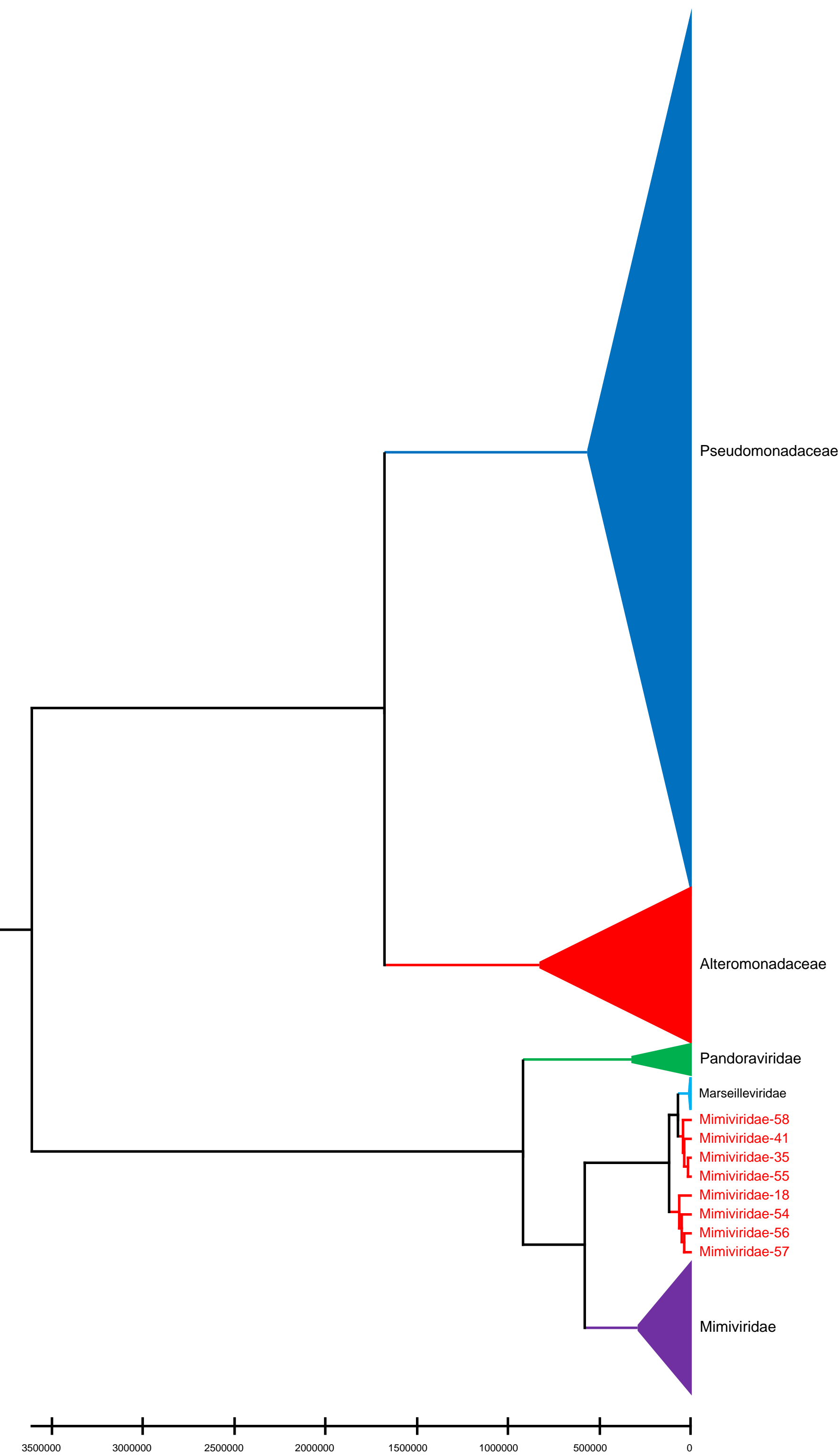

Supplement: Supplemental Information 13 [file peerj-10-13544-s013.pdf]

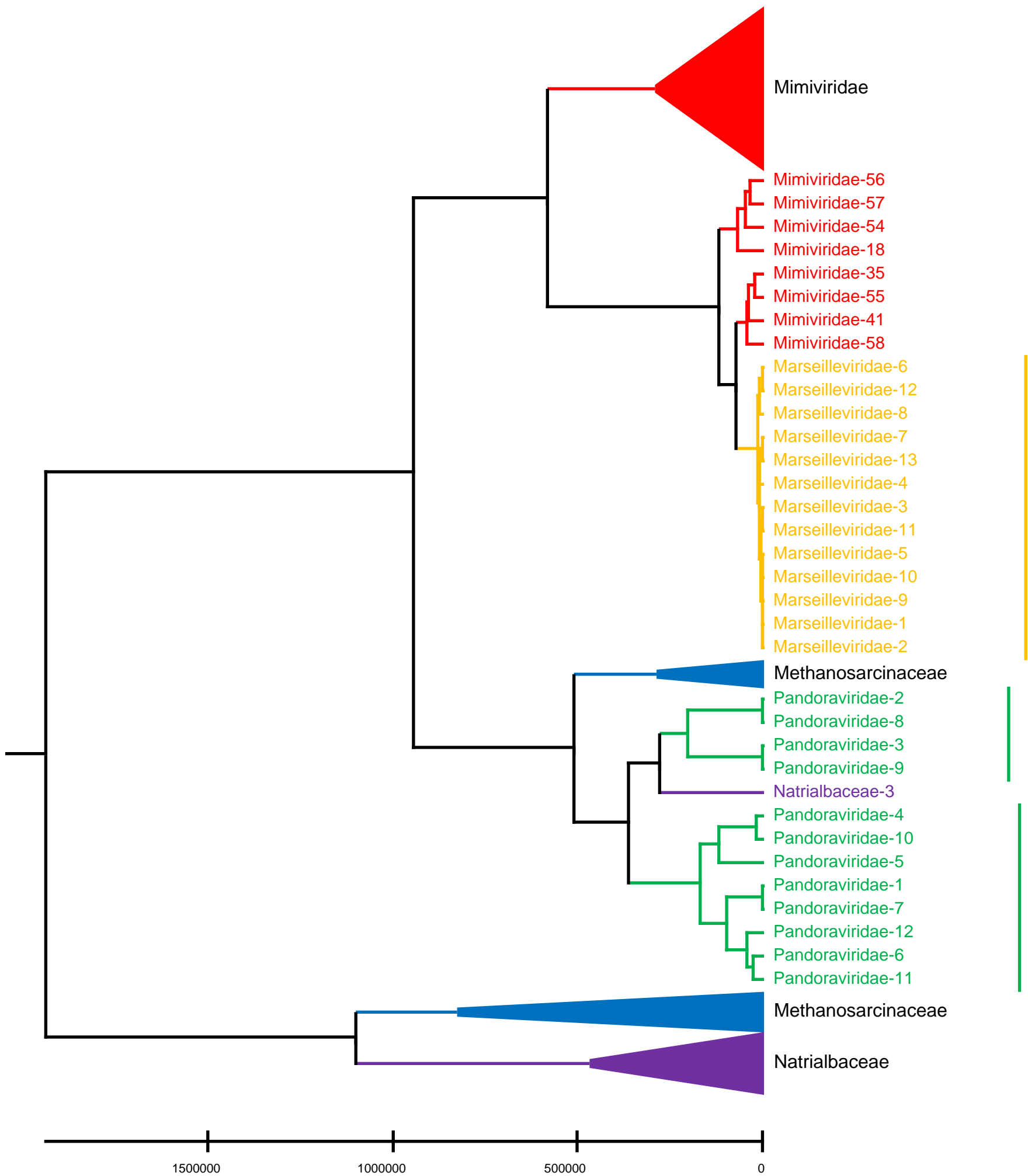

Supplement: Supplemental Information 14 [file peerj-10-13544-s014.pdf]

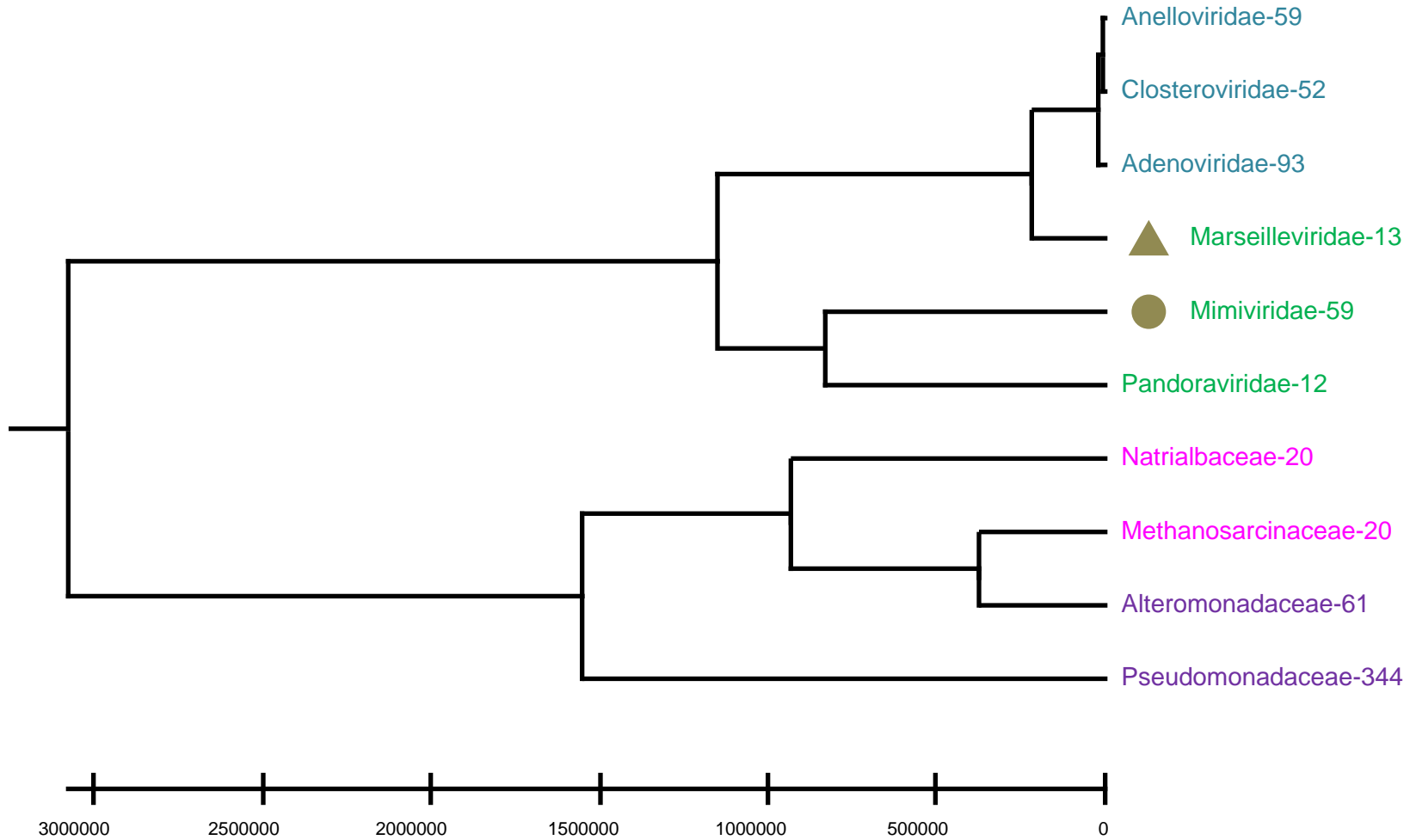

Supplement: Supplemental Information 15 [file peerj-10-13544-s015.pdf]

A

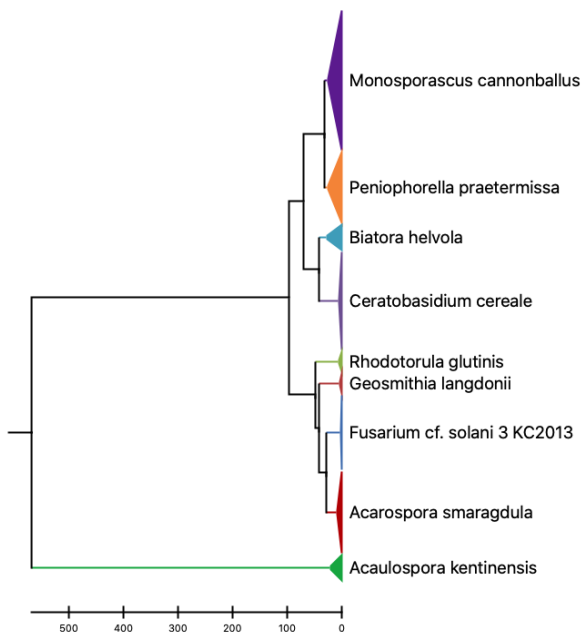

B

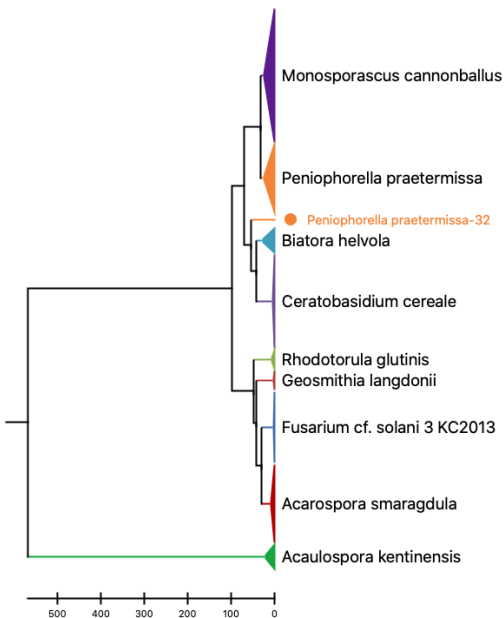

Supplement: Supplemental Information 16 — (A) Phylogenetic tree of nine species of fungi based on our method. (B) Phylogenetic tree of nine species of fungi based on the previous correlation method. [file peerj-10-13544-s016.pdf]
