# Supplementary material for: An efficient numerical representation of genome sequence: natural vector with covariance component
Source: PeerJ. 2022 Jun 16;10:e13544. doi: 10.7717/peerj.13544 (PMC9206847; doi:10.7717/peerj.13544)
Supplement: Supplemental Information 9 — We select a viral genome sequence (Accession number in GenBank is NC_035619, sequence length is 30620 bp) and search its similar sequences. (A) The parameters choice for the similar sequence search using BLAST (https://blast.ncbi.nlm.nih.gov/Blast.cgi). (B) Similar sequence search results using BLAST for the viral genome sequence (Accession number in GenBank is NC_035619). (C) The top ten similar sequences using our 18-dimensional natural vector method. Here the biological distance between two sequences is measured using the Euclidean distance of their corresponding 18-dimensional natural vectors, which is commonly used in our previous studies. It takes about 20 min for BLAST to get three similar sequences (Accession number are NC_002685.2, NC_020074.1, and NC_004037.2) for the target sequence, and the percentage identities are 73.05%, 72.75%, and 73.76%, respectively (Figure S9B). Using our 18-dimensional natural vector method, it takes about 0.024914 s to get the similar sequences from our extracted Virus dataset (Table 2 in the main text). The top three similar sequences are the same using BLAST and our method, but our method runs faster. In addition, there is a sequence length limitation for BLAST, so it is inconvenient to search similar sequences for the bacterial genomic sequence. But our methods can deal with these sequences efficiently. [file peerj-10-13544-s009.pdf]

A

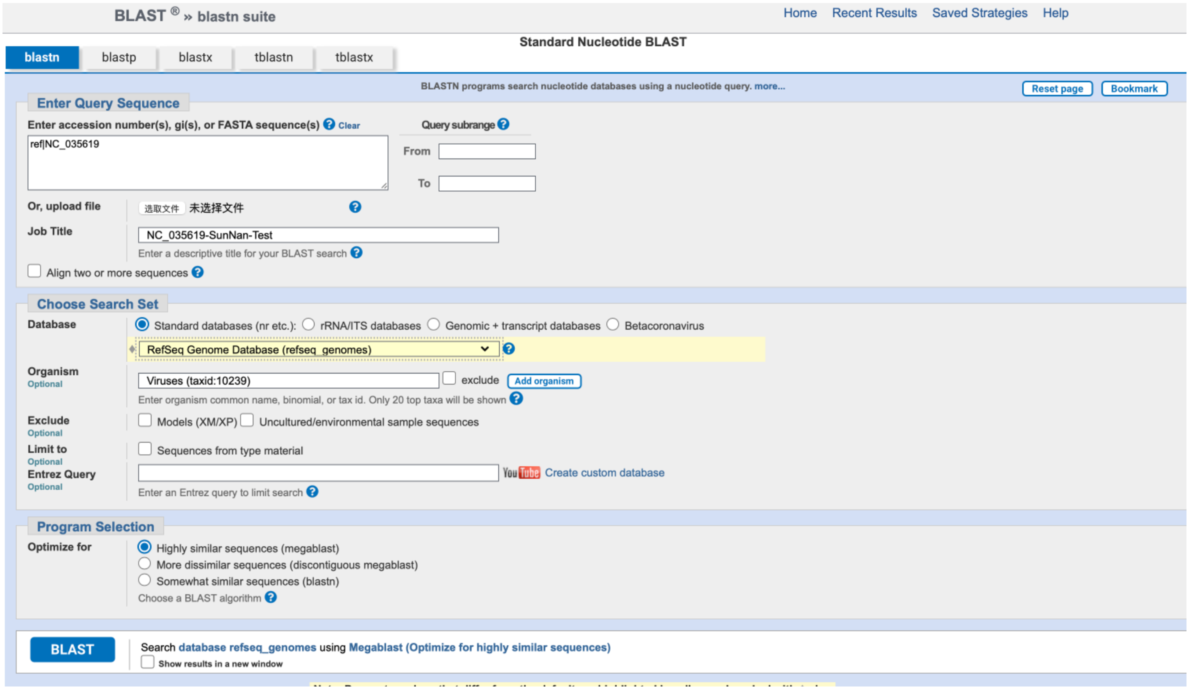

B

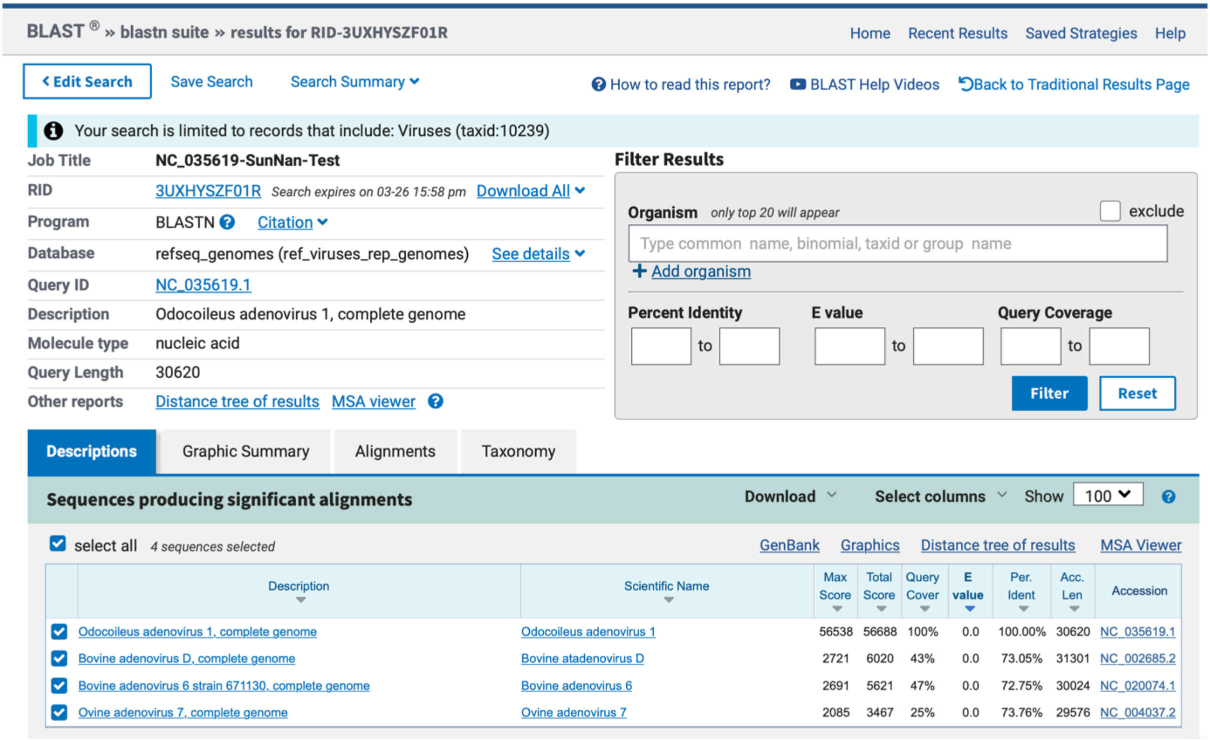

C

	Accession No.	Euclidean dis.	nA	nC	nG	nT	uA	uC	uG	uT	DA
	NC_035619	0	10524	5303	4874	9917	15995.78098	16160.73015	14473.50821	14538.61289	2511.227073
1	NC_002685	983.2994623	10318	5857	5149	9977	16443.2548	16260.30186	14850.49	14887.10905	2547.324948
2	NC_020074	1067.668091	9892	5463	5071	9598	15722.95309	15642.69724	14395.81404	14247.40779	2473.272774
3	NC_004037	1372.322754	10180	5374	4564	9458	15482.06906	15425.78936	14240.60955	13944.26718	2449.353633
4	NC_029314	1507.241747	10416	4789	4839	9719	14828.4351	15960.66047	13979.32445	14857.33429	2471.927443
5	NC_034626	1548.085262	10021	4965	5288	9649	15512.34208	15157.46747	14974.29917	14283.12012	2358.170358
6	NC_030860	1586.082317	9765	4848	5273	9695	15364.14798	15416.48288	13994.65598	14334.06251	2348.378978
7	NC_024150	1670.17358	10259	5668	5741	10041	16611.0732	16767.92855	15367.26598	14846.04143	2538.002638
8	NC_035072	1681.333264	9959	4929	5267	9657	15396.24029	15112.53479	15043.02962	14221.81837	2350.914822
9	NC_030874	1845.508421	10007	4483	4655	10017	15020.23334	15303.65983	14045.8073	14068.95158	2341.537692
10	NC_006635	2099.095441	9609	5100	5041	10124	15015.24175	14751.50686	14727.17139	15062.13562	2584.563516

	Accession No.	DC	DG	DT	Cov(A,C)	Cov(A,G)	Cov(A,T)	Cov(C,G)	Cov(C,T)	Cov(G,T)
	NC_035619	2562.074722	2509.361931	2550.075425	5356.36061	5397.777822	5062.3814	5078.172535	5360.782019	5396.681189
1	NC_002685	2653.842627	2568.854599	2608.437561	5380.465336	5420.651188	5155.463462	5239.015568	5437.770591	5476.38375
2	NC_020074	2509.422318	2509.814441	2472.209743	5192.980707	5251.350286	4946.061315	5022.701078	5170.087438	5225.481752
3	NC_004037	2467.028342	2411.341657	2451.234495	5163.59653	5272.647616	4903.83435	4899.208891	5111.504953	5203.741247
4	NC_029314	2433.106515	2329.418886	2554.479399	5295.362771	5214.396619	5026.56174	4762.051147	5347.013627	5263.89345
5	NC_034626	2624.921546	2616.577047	2472.656368	5197.855721	5147.016983	4829.525316	5243.838453	5329.964532	5277.1357
6	NC_030860	2542.056545	2539.497403	2467.149704	5124.049047	5061.89347	4815.132601	5085.932526	5286.494765	5218.190957
7	NC_024150	2691.304696	2638.151081	2654.28993	5414.958845	5366.259035	5191.343122	5329.224766	5554.861319	5505.116279
8	NC_035072	2621.483025	2616.403911	2454.800235	5185.928691	5135.419072	4804.685126	5240.598904	5308.89737	5256.217626
9	NC_030874	2448.948939	2407.17175	2492.808345	5137.519398	5074.933843	4834.422184	4856.194657	5364.568648	5297.732345
10	NC_006635	2437.702767	2402.739013	2466.533166	5323.587356	5308.661147	5049.736327	4840.727125	5205.359041	5190.927958
