# Supplementary material for: An efficient numerical representation of genome sequence: natural vector with covariance component
Source: PeerJ. 2022 Jun 16;10:e13544. doi: 10.7717/peerj.13544 (PMC9206847; doi:10.7717/peerj.13544)
Supplement: Supplemental Information 10 — Fifteen DNA barcode sequences of fungi are selected to construct the phylogenetic tree. (A) The phylogenetic tree using MUSCLE method (https://www.ebi.ac.uk/Tools/msa/muscle/). (B) The phylogenetic tree using our method. The algorithm to construct the phylogenetic tree is neighbor-joining (NJ), which is an approach based on distance to construct un-rooted tree. The distance matrix is calculated using the commonly used Euclidean distance. Using our method, the two families, Glomerellaceae and Strophariaceae can be separated better. The reason is that our method is based on the distribution of nucleotides from the perspective of statistic. Furthermore, since there are many bacterial genomic sequences in our study, MUSCLE can’t get the phylogenetic results in a reasonable time. Therefore, our method is better than MUSCLE in some sense. [file peerj-10-13544-s010.pdf]

A

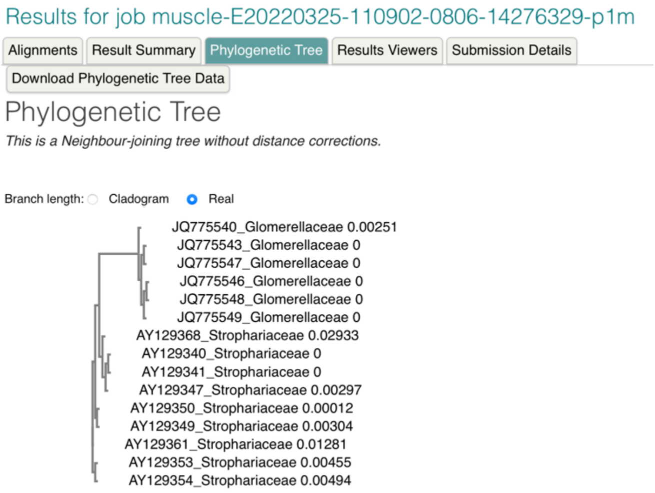
Results for job muscle-E20220325-110902-0806-14276329-p1m
Alignments
Result Summary
Phylogenetic Tree
Results Viewers
Submission Details
Download Phylogenetic Tree Data
Phylogenetic Tree
This is a Neighbour-joining tree without distance corrections.
Branch length:
Cladogram
Real
JQ775540_Glomerellaceae 0.00251
JQ775543_Glomerellaceae 0
JQ775547_Glomerellaceae 0
JQ775546_Glomerellaceae 0
JQ775548_Glomerellaceae 0
JQ775549_Glomerellaceae 0
AY129368_Strophariaceae 0.02933
AY129340_Strophariaceae 0
AY129341_Strophariaceae 0
AY129347_Strophariaceae 0.00297
AY129350_Strophariaceae 0.00012
AY129349_Strophariaceae 0.00304
AY129361_Strophariaceae 0.01281
AY129353_Strophariaceae 0.00455
AY129354_Strophariaceae 0.00494

B

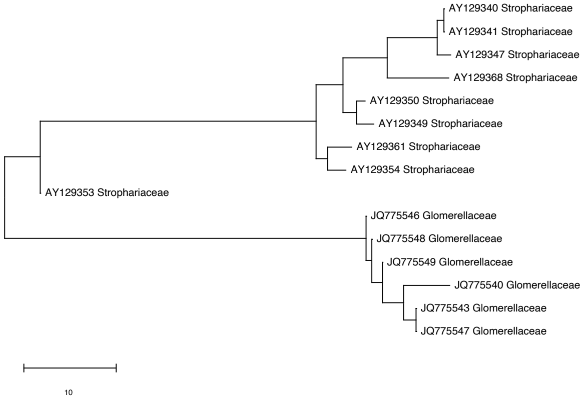
AY129340 Strophariaceae
AY129341 Strophariaceae
AY129347 Strophariaceae
AY129368 Strophariaceae
AY129350 Strophariaceae
AY129349 Strophariaceae
AY129361 Strophariaceae
AY129354 Strophariaceae
AY129353 Strophariaceae
JQ775546 Glomerellaceae
JQ775548 Glomerellaceae
JQ775549 Glomerellaceae
JQ775540 Glomerellaceae
JQ775543 Glomerellaceae
JQ775547 Glomerellaceae
10
