# Supplementary material for: An efficient numerical representation of genome sequence: natural vector with covariance component
Source: PeerJ. 2022 Jun 16;10:e13544. doi: 10.7717/peerj.13544 (PMC9206847; doi:10.7717/peerj.13544)
Supplement: Supplemental Information 12 [file peerj-10-13544-s012.pdf]

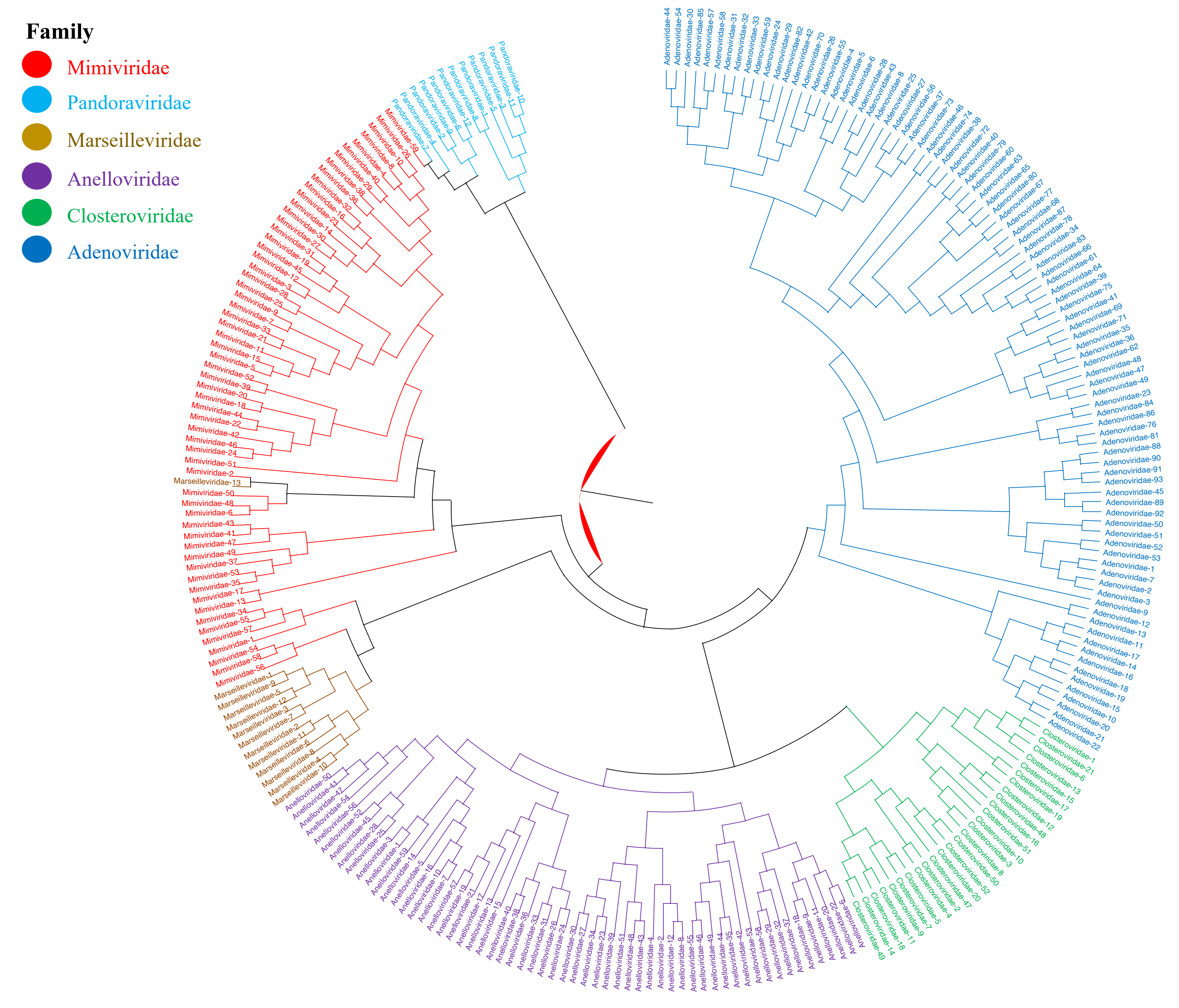
Family
Mimiviridae
Pandoraviridae
Marseilleviridae
Anelloviridae
Closteroviridae
Adenoviridae
