# Supplementary material for: An efficient numerical representation of genome sequence: natural vector with covariance component
Source: PeerJ. 2022 Jun 16;10:e13544. doi: 10.7717/peerj.13544 (PMC9206847; doi:10.7717/peerj.13544)
Supplement: Supplemental Information 17 [file peerj-10-13544-s017.docx]

| Family/group | Number of sequences | G+C contents (%) | | | | |
| --- | --- | --- | --- | --- | --- | --- |
|  |  | Maximum | Minimum | Mean | Median | Std |
| Pandoraviridae | 12 | 63.9 | 57.9 | 61.3 | 60.7 | 1.8 |
| Poxviridae | 39 | 63.4 | 17.8 | 29.9 | 29.8 | 9.3 |
| Phycodnaviridae | 82 | 60.2 | 25.0 | 41.6 | 40.8 | 6.6 |
| Iridoviridae | 10 | 47.9 | 27.2 | 31.7 | 29.5 | 6.2 |
| Ascoviridae | 3 | 45.9 | 35.2 | 40.0 | 38.9 | 5.4 |
| Marseilleviridae | 13 | 44.7 | 42.9 | 43.8 | 44.2 | 0.8 |
| Polydnaviridae | 5 | 43.2 | 33.6 | 37.7 | 36.7 | 4.3 |
| Pithoviridae | 3 | 35.8 | 35.4 | 35.7 | 35.8 | 0.2 |
| Mimiviridae | 59 | 29.4 | 21.2 | 26.3 | 25.5 | 1.9 |
