# Supplementary material for: An efficient numerical representation of genome sequence: natural vector with covariance component
Source: PeerJ. 2022 Jun 16;10:e13544. doi: 10.7717/peerj.13544 (PMC9206847; doi:10.7717/peerj.13544)
Supplement: Supplemental Information 18 [file peerj-10-13544-s018.docx]

| Virus species | Abbreviation | Segment | Accession | Genus | Max  Len | Min  Len | Average  Len | Total |
| --- | --- | --- | --- | --- | --- | --- | --- | --- |
| *Glypta fumiferanae ichnovirus* | GfIV | 105 | NC_008837-008941 | *Ichnovirus* | 5156 | 1533 | 2777.1 | 291597 |
| *Hyposoter fugitivus ichnovirus* | HfIV | 56 | NC_008946-009003 | *Ichnovirus* | 8851 | 2755 | 4394.5 | 246092 |
| *Cotesia congregata bracovirus* | CcBV | 30 | NC_006633-006662 | *Bracovirus* | 41573 | 4981 | 18922.3 | 567670 |
| *Campoletis sonorensis ichnovirus* | CsIV | 23 | NC_007985-008008 | *Ichnovirus* | 19557 | 6283 | 10460.7 | 240596 |
| *Microplitis demolitor bracovirus* | MdBV | 15 | NC_007028-007044 | *Bracovirus* | 34334 | 3611 | 12348.1 | 185221 |
