# Supplementary material for: An efficient numerical representation of genome sequence: natural vector with covariance component
Source: PeerJ. 2022 Jun 16;10:e13544. doi: 10.7717/peerj.13544 (PMC9206847; doi:10.7717/peerj.13544)
Supplement: Supplemental Information 19 [file peerj-10-13544-s019.docx]

| Label in phylogenetic tree | Virus name | SeqLen | Accession number |
| --- | --- | --- | --- |
| Mimiviridae-1 | *Cafeteria roenbergensis virus BV-PW1 (CroV)* | 617453 | GU244497 |
| Mimiviridae-2 | *Cafeteria roenbergensis virus BV-PW1 (CroV)* | 617453 | NC_014637 |
| Mimiviridae-18 | *Megavirus courdo7* | 529672 | JN885991 |
| Mimiviridae-35 | *Moumouvirus Monve* | 345413 | JN885998 |
| Mimiviridae-41 | *Catovirus CTV1* | 379946 | KY684084 |
| Mimiviridae-54 | *Hokovirus HKV1* | 450695 | KY684103 |
| Mimiviridae-55 | *Hokovirus HKV1* | 317278 | KY684104 |
| Mimiviridae-56 | *Hokovirus HKV1* | 407933 | KY684105 |
| Mimiviridae-57 | *Klosneuvirus KNV1* | 451653 | KY684108 |
| Mimiviridae-58 | *Klosneuvirus KNV1* | 333228 | KY684109 |
